# Liquid Extraction Surface Analysis Mass Spectrometry of ESKAPE Pathogens

**DOI:** 10.1021/jasms.0c00466

**Published:** 2021-03-01

**Authors:** Jana Havlikova, Robin C. May, Iain B. Styles, Helen J. Cooper

**Affiliations:** † EPSRC Centre for Doctoral Training in Physical Sciences for Health, University of Birmingham, Edgbaston, Birmingham B15 2TT, United Kingdom; ‡ School of Biosciences, University of Birmingham, Edgbaston, Birmingham B15 2TT, United Kingdom; § Institute of Microbiology and Infection, University of Birmingham, Edgbaston, Birmingham B15 2TT, United Kingdom; ∥ School of Computer Science, University of Birmingham, Edgbaston, Birmingham B15 2TT, United Kingdom

## Abstract

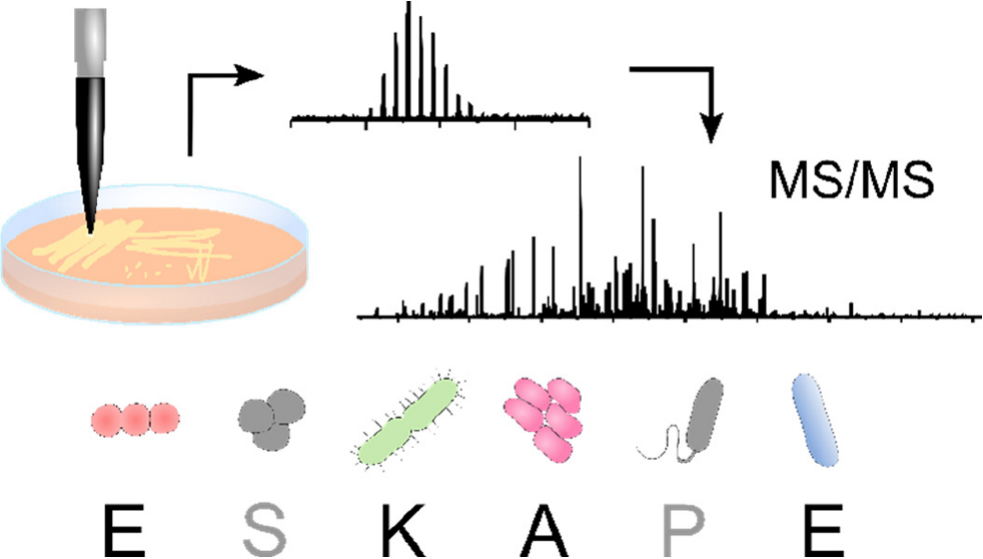

The ESKAPE pathogens (*Enterococcus faecium*, *Staphylococcus aureus*, *Klebsiella pneumoniae*, *Acinetobacter baumannii*, *Pseudomonas aeruginosa*, and *Enterobacter cloacae*) represent clinically important bacterial species that are responsible for most hospital-acquired drug-resistant infections; hence, the need for rapid identification is of high importance. Previous work has demonstrated the suitability of liquid extraction surface analysis mass spectrometry (LESA MS) for the direct analysis of colonies of two of the ESKAPE pathogens (*Staphylococcus aureus* and *Pseudomonas aeruginosa*) growing on agar. Here, we apply LESA MS to the remaining four ESKAPE species (*E. faecium* E745, *K. pneumoniae* KP257, *A. baumannii* AYE, and *E. cloacae* S11) as well as *E. faecalis* V583 (a close relative of *E. faecium*) and a clinical isolate of *A. baumannii* AC02 using an optimized solvent sampling system. In each case, top-down LESA MS/MS was employed for protein identification. In total, 24 proteins were identified from 37 MS/MS spectra by searching against protein databases for the individual species. The MS/MS spectra for the identified proteins were subsequently searched against multiple databases from multiple species in an automated data analysis workflow with a view to determining the accuracy of identification of unknowns. Out of 24 proteins, 19 were correctly assigned at the protein and species level, corresponding to an identification success rate of 79%.

## Introduction

The ESKAPE pathogens are six clinically relevant bacterial species *Enterococcus faecium*, *Staphylococcus aureus*, *Klebsiella pneumoniae*, *Acinetobacter baumannii*, *Pseudomonas aeruginosa*, and *Enterobacter* spp., of which two are Gram-positive (*E. faecium*, *S. aureus*) and the remainder are Gram-negative.^1^ The ESKAPE microbes are responsible for most hospital-acquired (nosocomial) infections,^2,3^ and their antibiotic resistance is rising.^4^ In fact, WHO reports at least 700 000 deaths annually due to infections by the drug-resistant strains,^5^ affecting both developed and developing countries.^6^ Development of improved tools for rapid and accurate identification of these bacteria and hence tailored treatment of patients is therefore of high importance.

The use of mass spectrometry (MS) for the analysis and identification of bacteria is well-established. Currently, the gold standard, FDA-approved mass spectrometry (MS) approach for identification of microbes is matrix-assisted laser desorption ionization time-of-flight (MALDI TOF) MS together with dedicated software for spectral fingerprinting.^7^ Nevertheless, MALDI TOF MS has some drawbacks for bacterial analysis including sample preparation requirements and the fact that analysis takes place under vacuum conditions, precluding analysis of live colonies. Ambient ionization MS techniques overcome these limitations.^8−16^ One such ambient technique is liquid extraction surface analysis (LESA) MS, a liquid microjunction sampling tool, based on diffusion of analytes into a droplet of solvent.^17^ The droplet is subsequently introduced into the mass spectrometer via chip-based nanoelectrospray (nanoESI).

We have been developing LESA MS as a tool for the top-down analysis of proteins directly from bacteria growing on solid substrates. This approach is different to the MALDI TOF MS diagnostic approach, because it focuses on identification of bacterial proteins rather than spectral matching. Another promising approach, which focuses on analysis of intact proteins, is liquid chromatography top-down proteomics (LC TDP). Recent work by Chamot-Rooke and co-workers demonstrated identification of >200 proteins and >500 proteoforms by LC TDP.^18^

Our pilot LESA MS study focused on *Escherichia coli* K12,^19^ while later work identified 39 proteins from multiple species including 2 of the ESKAPE pathogens *Staphylococcus aureus* and *Pseudomonas aeruginosa.*(20) More recent work focused on the analysis of bacteria growing on three-dimensional (3D) living skin equivalents, with the aim of simultaneous identification of bacterial and human skin proteins.^21^*In vitro* skin models were inoculated with three ESKAPE pathogens, *Klebsiella pneumoniae*, *Pseudomonas aeruginosa*, and two different strains of *Staphylococcus aureus*. In each case, LESA MS analysis showed that one to two bacterial proteins could be detected after 48 h and that both bacterial and human skin proteins were observed in the same mass spectra.

Here, we extend the top-down LESA MS approach to the remaining three ESKAPE pathogens—*Enterococcus faecium* E745 (and its close relative *Enterococcus faecalis* V583, commonly found in the hospital environment), *Acinetobacter baumannii* (the reference strain AYE and a clinical isolate AC02), and *Enterobacter cloacae* S11—as well as expanding the study of *Klebsiella pneumoniae* KP257 (previously only considered in the context of *in vitro* skin models). A key consideration if LESA MS is to find use as a diagnostic tool is a universal sampling approach, i.e., a single solvent system suitable for all Gram-positive and Gram-negative species. We first optimized the solvent system to enable successful protein extractions from all of the ESKAPE pathogens, before applying MS/MS for top-down protein identification. In total, 24 proteins were identified from 37 MS/MS spectra. Finally, we compare protein identification from searches against multiple databases and associated success of LESA diagnosis.

## Materials and Methods

### Materials

Analytical grade acetonitrile, water, and formic acid were purchased from Fisher Scientific (Loughborough, UK). Bacteriological agar was purchased from Appleton Woods (Birmingham, UK). LB broth (yeast extract (VWR, Lutterworth, UK), peptone (Sigma-Aldrich, Gillingham, UK) and sodium chloride (Fisher Scientific, Loughborough, UK)), BHI broth (dehydrated brain heart infusion (VWR, Lutterworth, UK)), LB agar (LB broth with added bacteriological agar), and BHI agar (BHI broth with added bacteriological agar) were prepared for culturing bacterial species. Bacterial strains *E. faecium* E745, *E. faecalis* V583, and *K. pneumoniae* KP257 were obtained from Willem van Schaik (Institute of Microbiology and Infection (IMI), University of Birmingham, UK), *S. aureus* MSSA476 and *P. aeruginosa* PS1054 were obtained from Mark Webber (Quadram Institute, Norwich, UK), *A. baumannii* AYE and AC02 were obtained from Jessica Blair (IMI, University of Birmingham, UK), and *E. cloacae* S11 was obtained from Allan McNally (IMI, University of Birmingham, UK).

### Preparation of Bacterial Colonies

Liquid cultures of each species were prepared. Approximately 1 μL of bacteria from the frozen glycerol stock was resuspended in 5–10 mL of liquid broth. Liquid cultures were incubated up to 18 h at shaking conditions (200 rpm) at 37 °C and plated on the solid agar media in 60 mm Petri dishes. Agar plates were incubated at static conditions and 37 °C overnight. ESKAPE pathogens were cultured in LB broth and LBA except for the *Enterococci* strains, which required culturing in BHI liquid broth and BHI agar. Two biological replicates were prepared for each species.

### LESA MS Analysis

LESA MS of proteins from bacterial colonies growing on agar plates was performed by use of the Advion Triversa Nanomate (Advion, Ithaca, NY) coupled to an Orbitrap Elite mass spectrometer (Thermo Fisher Scientific, Bremen, Germany) as described previously.^19,20^ For each biological replicate, between 1–11 technical replicates were performed. The optimized extraction solvent system (see Results and Discussion section) comprised acetonitrile, water, and formic acid (60:35:5). The agar plates were placed next to half of a 96-well microtiter plate in the Triversa Nanomate. The robotic pipet of the Triversa Nanomate aspirated 3 μL of the sampling solvent system and relocated to the new position above the sample. The descending pipet tip touched the colony surface and dispensed 2 μL of the solvent system. Subsequently, 2.5 μL of the solvent system containing analytes was reaspirated back to the pipet tip and introduced into the mass spectrometer via chip-based nanoESI at a gas pressure of 0.3 psi and a tip voltage of 1.75 kV. The Triversa Nanomate platform was controlled with the Chipsoft software 8.3.1. (Advion, Ithaca, NY, USA).

The mass spectra were acquired for (at least) 3 min in full scan positive ion mode with a mass range of 600–2000 *m*/*z* at a resolution of 120 000 at 400 *m*/*z* in the Orbitrap mass analyzer. The automatic gain control (AGC) target was 1 × 10^6^ charges. Each MS scan comprised a single microscan. Precursor ions were selected for fragmentation with an isolation window of 3 *m*/*z*. Collision-induced dissociation (CID) was performed in the ion trap with use of helium gas at a normalized collision energy of 35%. The AGC target was between 5 × 10^4^ and 1 × 10^5^ charges. MS/MS mass spectra were recorded in the Orbitrap for (at least) 5 min at a resolution of 120 000 at 400 *m*/*z*, and each scan comprised 30 coadded microscans.

### Data Analysis and Identification of Proteins

Top-down identification of proteins from fragmentation mass spectra was achieved by use of the ProSight 4.1 software (Thermo Fisher Scientific, Bremen, Germany). The whole organism proteome databases were downloaded in XML format from the UniProt Web site (uniprot.org) for *E. faecium* ATCC BAA-472/TX0016/DO (UP000005269, 3059 entries, 15 638 proteoforms), *E. faecalis* ATCC 700802/V583 (UP000001415, 3240 entries, 17,469 proteoforms), *S. aureus* NCTC8325 (UP000008816, 2889 entries, 14 793 proteoforms), *K. pneumoniae* ATCC 700721 (UP000000265, 5126 entries, 26 531 proteoforms), *A. baumannii* AYE (UP000002446, 3652 entries, 18 616 proteoforms), *P. aeruginosa* ATCC 15692/PA01 (UP000002438, 5563 entries, 29 775 proteoforms), and *E. cloacae* S611 (UP000017834, 3989 entries, 20 832 proteoforms). Each database was constructed as a standard top-down database, taking into account cleavage of initial methionines, N-terminal acetylation, and N-terminal formylation. Single-nucleotide polymorphisms (SNPs) and post-translational modifications (PTMs) were considered with a maximum of 13 features per sequence and maximum mass of 70 kDa. The MS/MS spectra were deconvoluted using the THRASH algorithm in the ProSight import profile window with default parameters and an S/N set to 2 or 3 depending on the quality of acquired MS/MS mass spectra. The absolute mass search included a delta-mass (Δ*m*) option for locating possible unknown post-translational modifications and mutations. The search window width was set to 1000 Da with a fragment tolerance of ±15 ppm and the minimum matching fragments number set to 4. Identified protein sequences were checked with the Sequence Gazer function of ProSight software followed by manual peak assignment where the fragment tolerance was narrowed to ±5 ppm.

BLAST (Basic Local Alignment Search Tool) searches were performed as follows: For each protein identified, the protein sequence was downloaded from Uniprot in FASTA format and searched against the nonredundant protein sequences (nr) database using the BLASTP algorithm with default parameters (blast.ncbi.nlm.nih.gov).

## Results and Discussion

### Optimization of Extraction Solvent System for LESA MS Analysis

**Figure 1:**
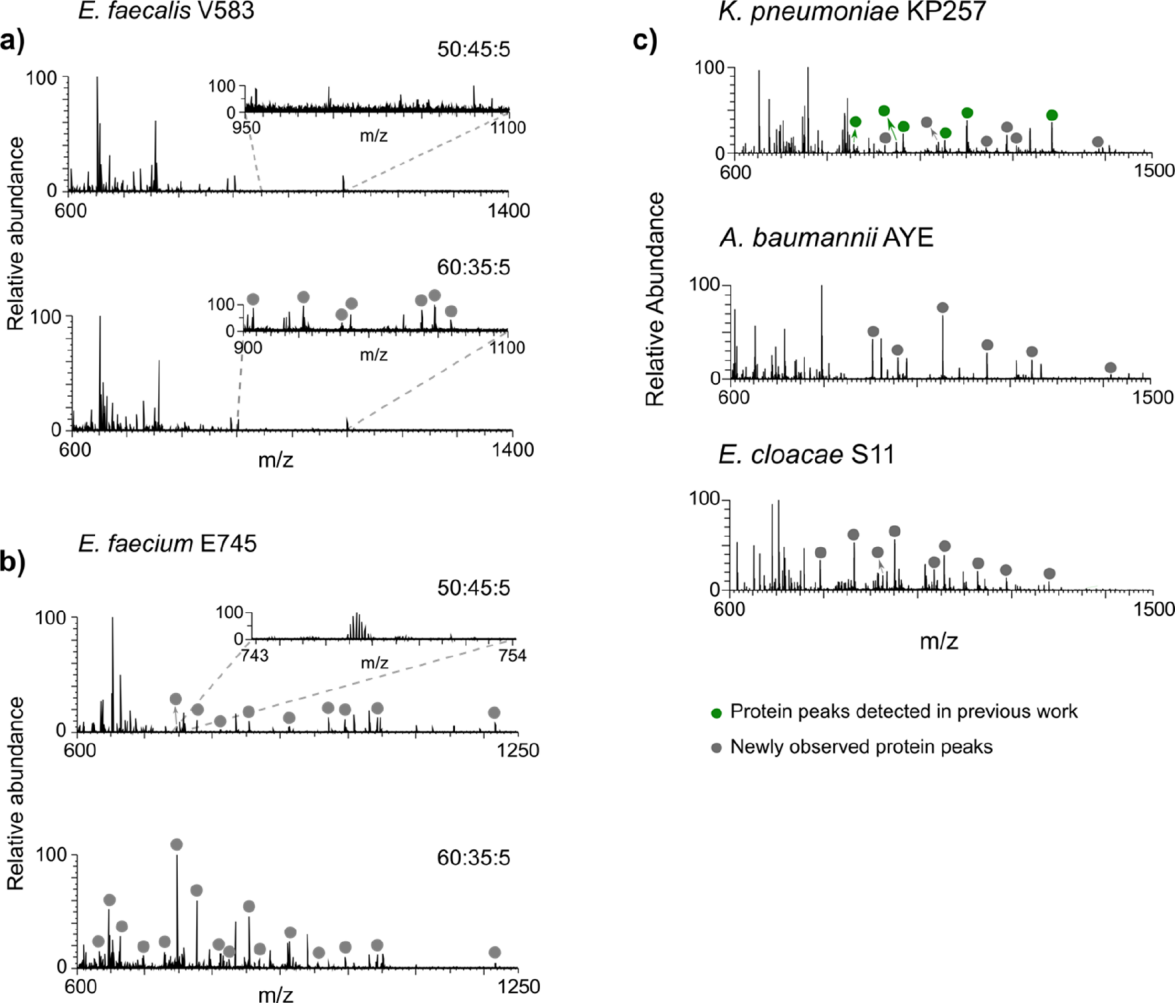
LESA mass spectra of bacterial colonies. Comparison of extraction solvent systems, acetonitrile/water/formic acid 50:45:5 and 60:35:5 for (a) *E. faecalis* V583 and (b) *E. faecium* E745. (c) LESA MS analysis of *K. pneumoniae* KP257, *A. baumannii* AYE, and *E. cloacae* S11 with the 60:35:5 extraction solvent system.

In our earlier work, two different solvent extraction systems comprising acetonitrile, water, and formic acid were employed for the LESA MS analysis of Gram-positive (50:45:5) and Gram-negative (40:59:1) bacteria.^20^ The higher acetonitrile and formic acid content for the Gram-positive species ensures the extraction of proteins, possibly due to cell lysis or increased ionization efficiency.^20^ Utilization of two extraction solvents is, however, impractical when analyzing multiple and unknown bacterial species. In addition, initial LESA MS experiments with the 50:45:5 extraction solvent system of Gram-positive *Enterococci* species resulted in the observation of no protein peaks for *E. faecalis* V583 and only a few, low abundance protein peaks for *E. faecium* E745 (Figure 1a,b). Subsequent experiments focused on *E. faecalis* V583 as a model organism for the optimization of extraction solvent composition. Solvents comprising various ratios of ethanol, acetonitrile, water, and formic acid were investigated (see Supporting Information, Figure S1). The best results, as determined by detection of the greatest number of protein peaks, were achieved with the 60:35:5 acetonitrile/water/formic acid solvent system (Figure 1a). (Interestingly, there appears to be a limit on the acetonitrile content: At 80% acetonitrile, no protein peaks were detected, likely a result of poor protein solubility). The optimized solvent system also resulted in detection of a greater number of proteins from *E. faecium* E745 (Figure 1b). The optimized LESA extraction solvent system was subsequently tested for the remaining three ESKAPE species that are the focus of this work (Figure 1c) as well as the previously studied *S. aureus* MSSA476 and *P. aeruginosa* PS1054 (Figure S2, Supporting Information). Representative LESA mass spectra from biological replicates are shown in Figure S3, Supporting Information. The results show that 60:35:5 acetonitrile/water/formic acid is suitable as a solvent for the extraction of proteins from both Gram-positive and Gram-negative ESKAPE microorganisms.

### LESA MS Analysis of ESKAPE Pathogens

**Figure 2:**
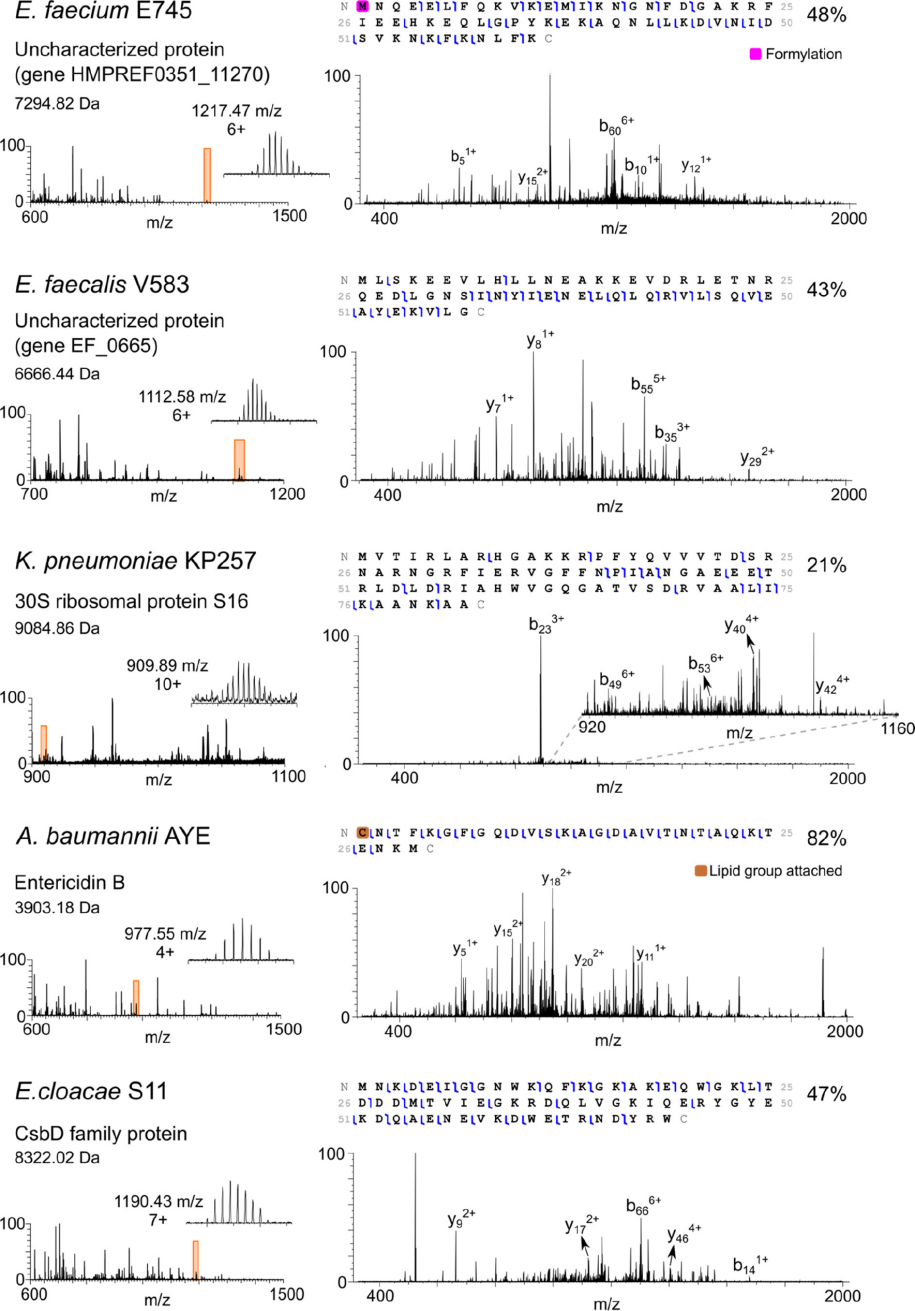
Representative MS/MS spectra corresponding to five newly identified proteins for the various ESKAPE species.

For each of the species studied, the most abundant protein peaks were selected for fragmentation by CID. The resulting MS/MS spectra were searched against the corresponding individual bacterial protein database. Top-down LESA MS analysis of the ESKAPE species resulted in identification of 24 proteins from 37 MS/MS spectra. MS/MS spectra and fragment assignments for all proteins are shown in the Supporting Information, File S1. Representative MS/MS spectra for the various species are shown in Figure 2.

### *Enterococci*

For *E. faecium* E745, five proteins were identified after CID fragmentation of 11 MS/MS spectra (see File S1, Supporting Information): 50S ribosomal protein L29, 30S ribosomal protein S20, and three uncharacterized proteins HMPREF0351_11703, HMPREF0351_11270, and HMPREF0351_12038. Four out of five of the identified proteins were observed in both biological replicates. According to Uniprot, the predicted HMPREF0351_11703 protein sequence includes a signal peptide. Our data suggest no signal peptide cleavage and formylation of the N-terminal methionine. The second uncharacterized protein HMPREF0351_11270 (see Figure 2) was identified with formylated methionine at the N-terminus, a modification so far unrecorded in the Uniprot database. For the third uncharacterized protein HMPREF0351_12038, a mutation R → Q was detected at either position 38 or position 43; however, no fragments were observed in this region to allow unambiguous localization.

A close relative of *E. faecium* is *E. faecalis*, a clinically important species associated with infective endocarditis, biofilm formation, and antimicrobial resistance.^22^ We chose to investigate this microbe due to its abundance in the hospital environment and increasing antibiotic resistance.^23^ LESA MS/MS of five *E. faecalis* V583 intact precursor ions resulted in the identification of four proteins (see File S1, Supporting Information): DNA-binding protein HU, 50S ribosomal protein L29, UPF0337 protein EF_1180, and uncharacterized protein (gene EF_0665). The UPF0337 protein EF_1180 is a protein inferred from homology and belongs to the bacterial general stress response protein (CsbD) family, while the uncharacterized protein (gene EF_0665) (see Figure 2) is a predicted protein. Comparison of the LESA mass spectra obtained from *E. faecalis* V583 and *E. faecium* E745 revealed that the 50S ribosomal protein L29 was observed for both species (Figure S4). The sequence of the protein from the two species differs by an N → K substitution at position 62 resulting in a mass difference Δ*m* = 14.07 Da. This observation is potentially useful as a diagnostic for differentiation between these two microorganisms.

### *Klebsiella pneumoniae*

Previous LESA MS experiments have only considered *Klebsiella pneumoniae* in the context of its growth on 3D living skin equivalents. In that work, two proteins were identified. A more in-depth LESA MS/MS analysis of the bacteria cultured on agar plates is therefore warranted. LESA MS/MS of nine precursor proteins from *K. pneumoniae* KP257 resulted in identification of six proteins, two of which (DNA-binding protein HU-α and KPN_00497) were identified in the previous analysis of the *in vitro* 3D skin models.^21^ All six proteins were observed in both biological replicates. The same protein mutation R → K at the position 49 and a signal peptide cleavage (1–19) was observed for the KPN_00497 protein. The four novel proteins (see File S1, Supporting Information) included two ribosomal proteins (50S ribosomal protein L29 and 30S ribosomal protein S16 (see Figure 2), one uncharacterized protein (gene yciG), and CsbD domain-containing protein. The search results indicated a signal peptide cleavage of the first 19 amino acids of the CsbD protein sequence previously unrecorded in the Uniprot database.

### *Acinetobacter baumannii*

LESA MS analysis of the Gram-negative reference strain *A. baumannii* AYE yielded mass spectra with highly abundant protein peaks (Figures 1c and Figure S5). Subsequent CID fragmentation of six intact precursors resulted in four protein identifications—three uncharacterized proteins (genes ABAYE1298, ABAYE2274, and ABAYE1876) and bacteriolytic lipoprotein entericidin B (see File S1, Supporting Information). All four proteins were observed in both biological replicates. For ABAYE2274, cleavage of the initial methionine was detected from the MS/MS data. The amino acid sequence of ABAYE1876 contains a signal peptide (1–14), information not yet recorded in the Uniprot database. Entericidin B is the first lipoprotein to be identified by LESA MS (see Figure 2). Lipoproteins are important for bacterial physiology as well as virulence and as activators of the host innate immune response.^24^ Bacterial lipoproteins are characterized by a conserved N-terminal lipid-modified cysteine residue. In this case, a mass shift of 813.72 Da was detected; however, the exact structure of the lipid group attached to the N-terminus remains unknown and would require further analysis. Despite the mass shift, there is high confidence in the protein assignment due to the high sequence coverage (82%) obtained. *A. baumannii* AYE was compared to the clinical strain *A. baumannii* AC02 (Figure S5). Again, LESA MS resulted in detection of several highly abundant protein peaks (Figure S5); however, their identification proved challenging. Six MS/MS spectra were searched against the AYE database; however, no protein IDs were assigned, suggesting dissimilarity in the protein amino acid sequences of these strains and a requirement for a new database. The dissimilarity can also be observed from the comparison of the AYE and AC02 mass spectra (Figure S5).

### *Enterobacter cloacae*

Mass spectra obtained following LESA of *E. cloacae* S11 contained many abundant peaks corresponding to proteins (Figure 1c). LESA MS/MS analysis of six intact precursors resulted in identification of five proteins (see File S1, Supporting Information)—50S ribosomal protein L29, DNA-binding protein, CsbD family protein (see Figure 2), UPF0391 membrane protein, and DUF1471 domain-containing binding protein. Four out of five proteins were detected in both biological replicates. The representative mass spectra shown in Figure S3, Supporting Information contain peaks corresponding to four and three of those proteins. For the UPF0391 membrane protein a new PTM—formylation at the N-terminus, not yet reported in the Uniprot database—was revealed. The DUF1471 protein sequence contains a signal peptide (1–21) and a mutation (E → N) at one of two potential positions—either 37 or 45; however, no fragments were observed in this region to allow unambiguous localization.

### Identification of ESKAPE Pathogens from Multiple Protein Databases

Initially, our goal was to investigate ESKAPE pathogens by top-down LESA MS combined with searching of individual species databases. If LESA MS is to find use as a diagnostic tool, however, correct identification of proteins (and species) from multiple databases is necessary. To evaluate that, a data analysis workflow was constructed in the ProSightPC software, in which each MS/MS mass spectrum was searched against all six individual ESKAPE protein databases (including *S. aureus* and *P. aeruginosa*) (i.e., an automated concurrent search of individual databases) using the absolute mass search function. The 24 MS/MS spectra corresponding to the newly identified proteins described above were used for the searches. The lowest e-score value was used as the indicator of protein assignment, and that assignment was compared with the known protein ID. The results are summarized in Table S1, Supporting Information.

In total, 19/24 proteins were correctly assigned, both in terms of protein ID and bacterial species. For two MS/MS spectra (corresponding to CscD domain-containing protein (*K. pneumoniae* KP257) and uncharacterized protein (gene ABAYE1876) (*A. baumannii* AYE)), no protein assignments were returned. Both CscD domain-containing protein and uncharacterized protein (gene ABAYE1876) were identified above using the biomarker search function in the ProSightPC software. That function is designed for identification of truncated proteins; therefore, the absolute mass search used in this automated data analysis workflow did not find any matching sequences.

Three proteins were misassigned, all from *E. cloacae* S11: DUF1471 domain-containing protein (*E. cloacae* S11) was assigned as an uncharacterized protein EF_2117 (*E. faecalis*), 50S ribosomal protein L29 was identified as the same protein but from *K. pneumoniae*, and UPF0391 membrane protein SAMEA2054040_04753 was assigned to UPF0391 membrane protein KPN_04833, again from *K. pneumoniae*. The DUF1471 domain-containing protein was identified above by use of a biomarker search within the ProSightPC software, and the misassignment appears to be the result of the absolute mass search. Both *Enterobacter* and *Klebsiella* belong to the family of Enterobacteriaceae; therefore, similarities in protein amino acid sequences between these species might be observed.

The overall success rate was 79% when both Gram-positive and Gram-negative species are considered. Generally, the accuracy of assignment of Gram-negative species is higher than for Gram-positives, which is also observed in MALDI TOF MS.^25^ Identification success rates for MALDI TOF MS at the species level vary between 84.1–94.9%^26,27^ for aerobic bacteria and routine isolates and 81.8% for both aerobes and anaerobes.^25,27^ Suggested improvements for LESA MS include the use of multiple databases at the genus and strain level, which might increase the success rate of protein identification in the future.

To further address the question of correct species identification, a BLAST search of the 24 identified proteins was performed to determine the specificity of the protein sequences. All of the protein sequences identified for *E. faecium*, *E. faecalis*, *K. pneumoniae*, and *A. baumannii* were unique to their species. Two out of the five *E. cloacae* proteins were species-specific, whereas DNA-binding protein HU shared a 100% sequence homology with *Cedecea davisae*, CsbD family protein shared a 100% sequence homology with *E. hormaechei*, and UPF0391 membrane protein shared 100% sequence homology with *Lelliotia amnigena*.

## Conclusion

The results show that a LESA MS sampling solvent system comprising 60:35:5 acetonitrile/water/formic acid is capable of extracting proteins from both Gram-positive and Gram-negative ESKAPE pathogens. Four out of six ESKAPE microbes *E. faecium*, *K. pneumoniae*, *A. baumannii*, and *E. cloacae*, including the clinically important strain *E. faecalis* and the clinical isolate *A. baumannii*, were investigated by a top-down LESA MS/MS method. The MS/MS mass spectra searches resulted in identifications of 24 proteins. The 50S ribosomal protein L29 was observed in three of the four ESKAPE species studied here. This protein has also previously been identified in the LESA mass spectra obtained from the ESKAPE pathogen *P. aeruginosa*.^20^ This result suggests that this protein may potentially serve as a biomarker for these species. For top-down LESA MS to be useful in bacterial identification, there is a requirement for proteins (and species) to be correctly identified following searching of MS/MS spectra against databases from multiple species. In this work, the overall identification success rate was determined to be 79%. *E. cloacae* presented the biggest challenge in terms of species identification based on results from both the multidatabase search and the BLAST search. Further development of LESA MS as a diagnostic requires application to a broader range of species. Nevertheless, the results presented suggest LESA MS has potential as a useful tool in clinical microbiology.

## Supporting Information Available

LESA extraction solvent system optimization (Figure S1); LESA MS of *S. aureus* and *P. aeruginosa* (Figure S2); LESA MS of biological replicates (Figure S3); comparison of LESA MS of enterococci 50S ribosomal protein L29 (Figure S4); comparison of LESA MS of *A. baumannii* strains (Figure S5); summary of results from multiple-database search (Table S1); protein assignments (File S1) (PDF)
