# Supplementary material for: Liquid Extraction Surface Analysis Mass Spectrometry of ESKAPE Pathogens
Source: J Am Soc Mass Spectrom. 2021 Mar 1;32(6):1345–51. doi: 10.1021/jasms.0c00466 (PMC8176453; doi:10.1021/jasms.0c00466)
Supplement: Supplementary file 1 — js0c00466_si_001.pdf [file js0c00466_si_001.pdf]

Supporting Information

**Liquid extraction surface analysis mass spectrometry of ESKAPE pathogens**

Jana Havlikova[1,2], Robin C. May[2,3], Iain B. Styles[4] and Helen J. Cooper[2*]

[1]EPSRC Centre for Doctoral Training in Physical Sciences for Health, University of Birmingham, Edgbaston, Birmingham, B15 2TT, United Kingdom

[2]School of Biosciences, University of Birmingham, Edgbaston, Birmingham, B15 2TT, United Kingdom

[3]Institute of Microbiology and Infection, University of Birmingham, Edgbaston, Birmingham, B15 2TT, United Kingdom

[4]School of Computer Science, University of Birmingham, Edgbaston, Birmingham, B15 2TT, United Kingdom

*Corresponding Author:
Helen J. Cooper
School of Biosciences, University of Birmingham, Edgbaston, B15 2TT, United Kingdom
h.j.cooper@bham.ac.uk

File S1: Protein assignments

Fig S1: LESA mass spectra of *E. faecalis* V583 sampled with the 5 different acetonitrile-based extraction solvent systems.

Fig S2: Mass spectra of *P. aeruginosa* PS1054 and *S. aureus* MSSA476 after LESA extraction with the optimized extraction solvent system (60:35:5 acetonitrile:water:formic acid).

Fig. S3: Representative LESA mass spectra from biological replicates of the four ESKAPE species.

Fig S4: *E. faecalis* V583 and *E. faecium* E745 LESA mass spectra showing the mass difference between the 50S ribosomal proteins L29.

Fig S5: Comparison of the *A. baumannii* AYE (reference strain) to the *A. baumannii* AC02 (clinical strain).

Table S1: Protein ID assignments after searches against all six individual ESKAPE databases.

**LESA extraction solvent system optimisation process**

Five different LESA extraction solvent systems were investigated. All of the solvent systems comprised acetonitrile, water and formic acid while one also included ethanol. The most suitable ratio for extracting proteins from colonies of *E. faecalis* V583 was shown to be 60:35:5 (acetonitrile:water:formic acid) (Fig. S1).

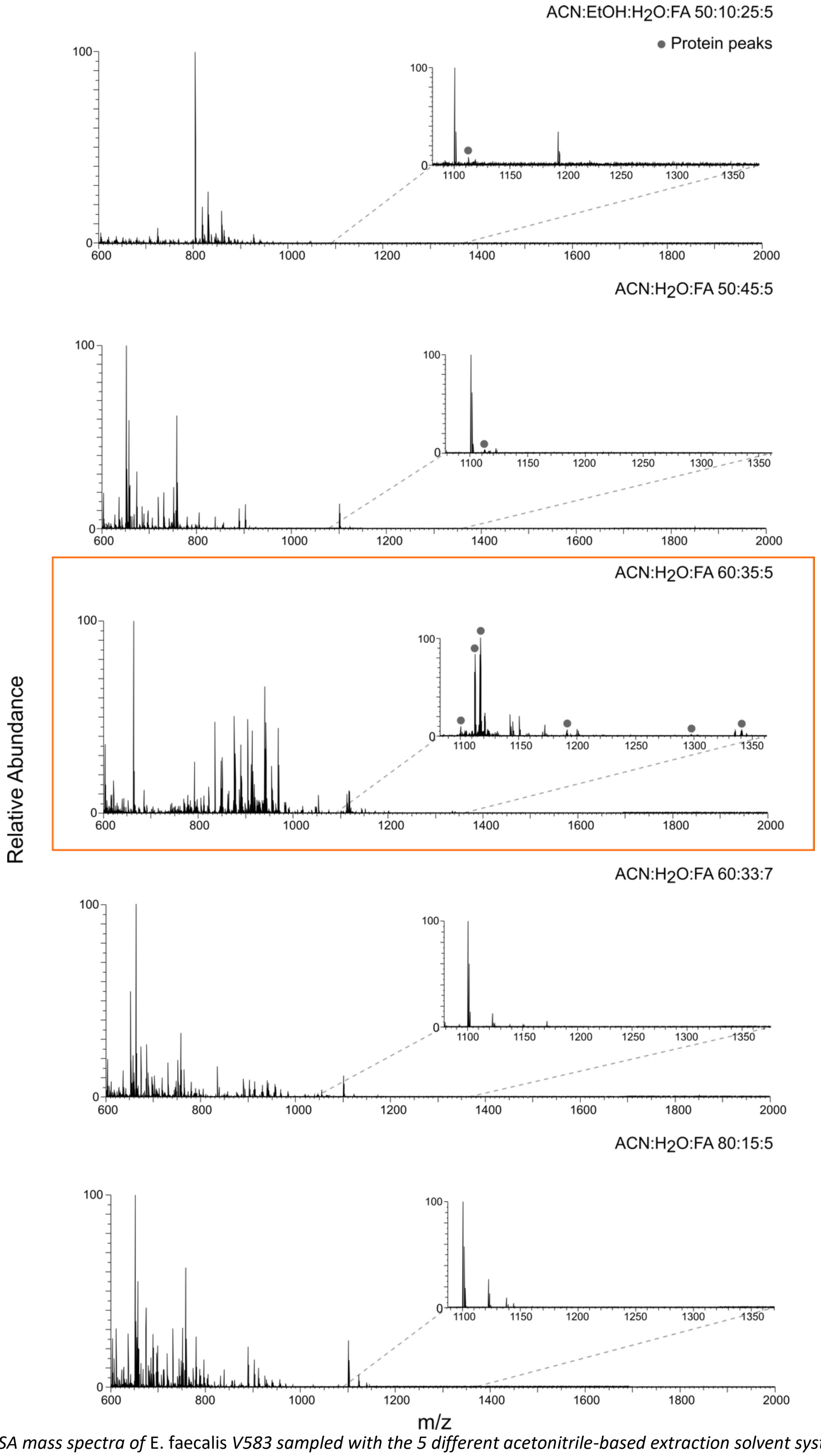


*Fig S 1 LESA mass spectra of* E. faecalis *V583 sampled with the 5 different acetonitrile-based extraction solvent systems. The solvent system resulting in the detection of the greatest number of protein peaks in the mass spectra is highlighted.*

**LESA extraction of proteins from *S. aureus* MSSA476 and *P. aeruginosa* PS1054 with the optimised solvent system**

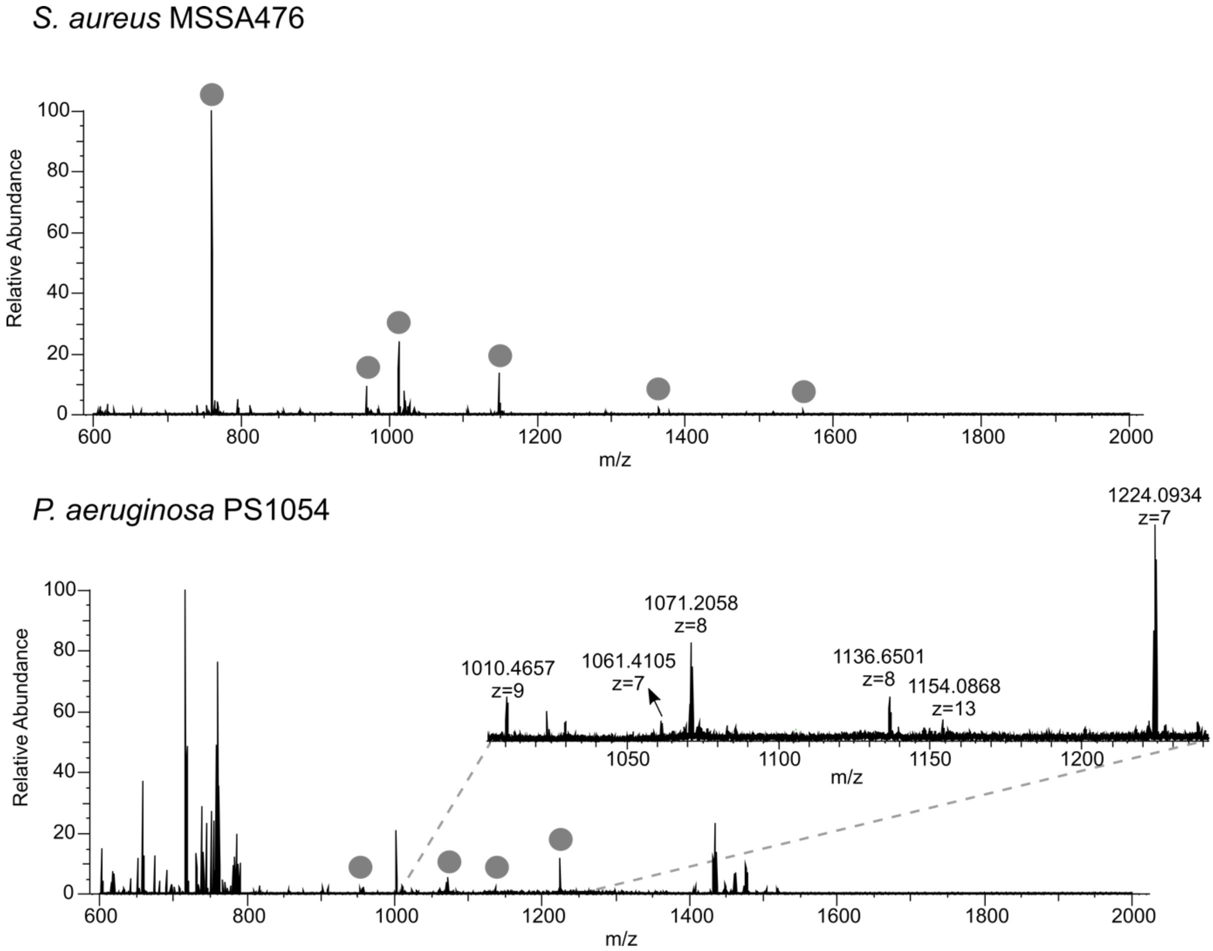


*Fig S 2 Mass spectra of P. aeruginosa PS1054 and S. aureus MSSA476 after LESA extraction with the optimized extraction solvent system (60:35:5 acetonitrile:water:formic acid).*

**Biological replicates of the four investigated ESKAPE pathogens**

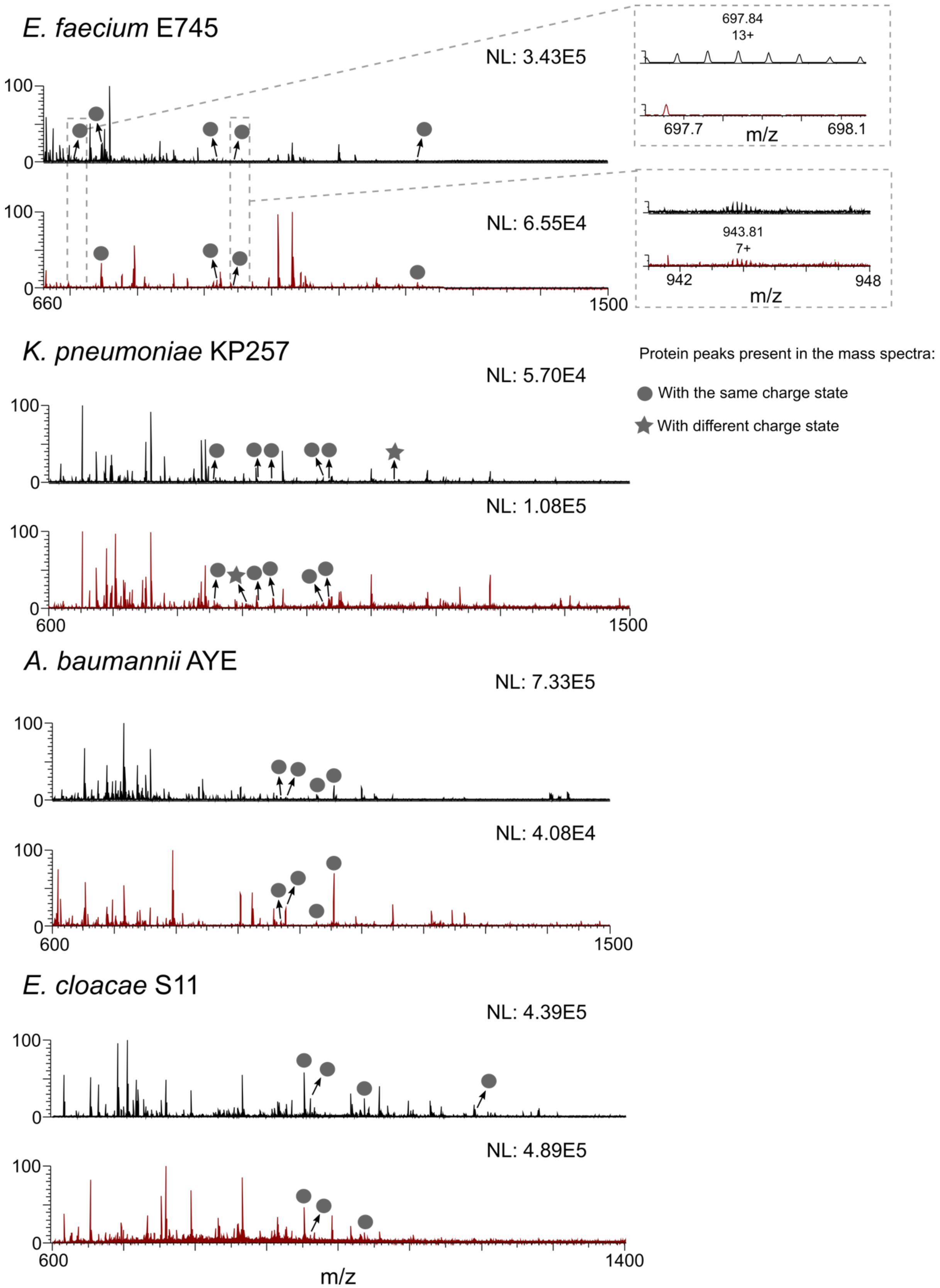


*Fig S 3 Representative mass spectra of biological replicates of the four investigated ESKAPE species.*

## Comparison of the Enterococci strains 50S ribosomal protein L29

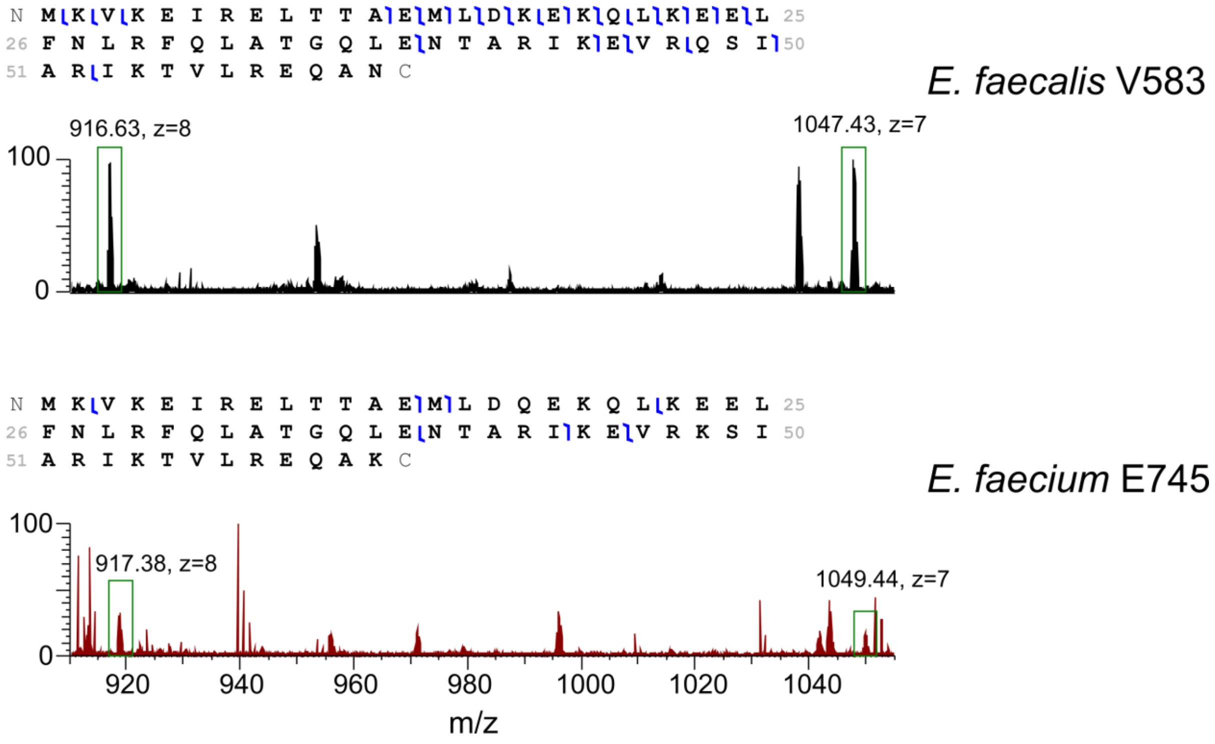


*Fig S 4 E. faecalis and E. faecium LESA mass spectra showing the mass difference between the 50S ribosomal proteins L29.*

**Comparison of *A. baumannii* reference and clinical strain**

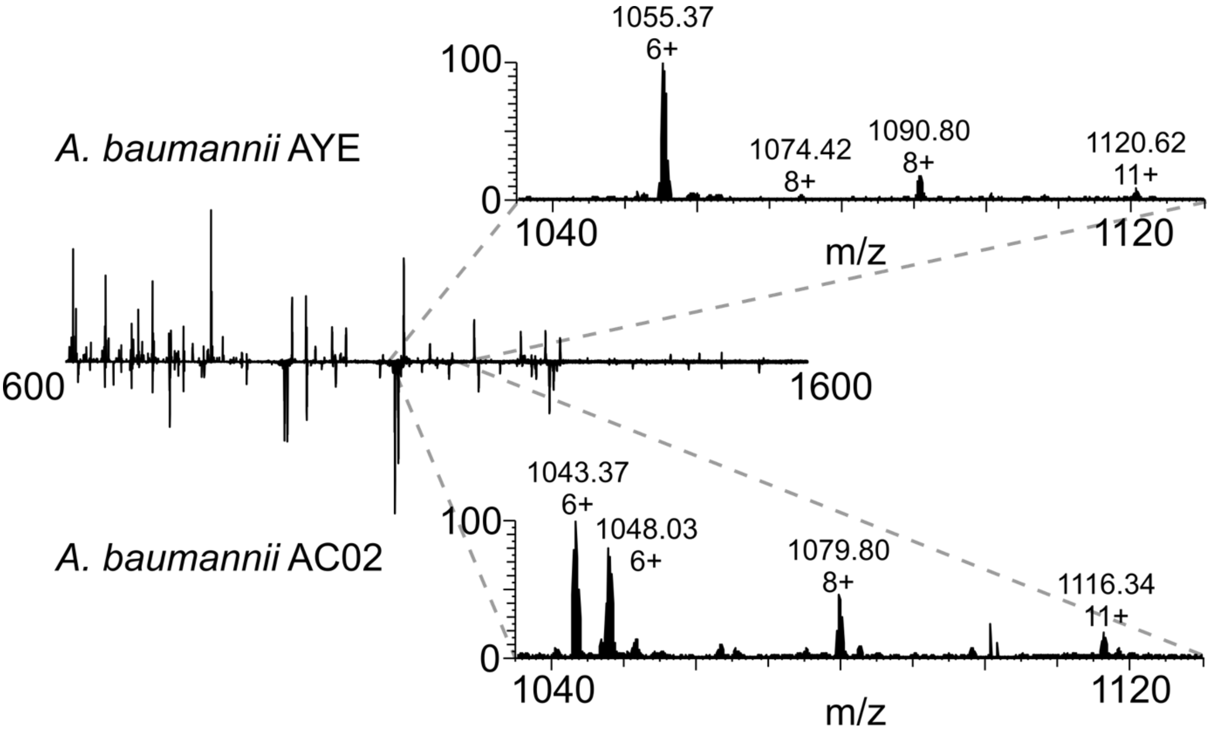


*Fig S 5 Comparison of the A. baumannii AYE (reference strain) to the A. baumannii AC02 (clinical strain).*

*Tab S 1 Protein ID assignments after searches against all six individual ESKAPE databases.*

| Species | Protein ID | Lowest e-score | Database assignment | Correct assignment? |
|---|---|---|---|---|
| *E. faecium* E745 | Uncharacterized protein Gene HMPREF0351_11703 | $3.02\times10^{-34}$ | Uncharacterized protein Gene HMPREF0351_11703 | yes |
| | Uncharacterised protein (gene HMPREF0351_11270) | $4.20\times10^{-07}$ | Uncharacterised protein gene HMPREF0351_11270 | yes |
| | 50S ribosomal protein L29 | $2.83\times10^{-10}$ | 50S ribosomal protein L29 | yes |
| | 30S ribosomal protein S20 | $4.40\times10^{-28}$ | 30S ribosomal protein S20 | yes |
| | Uncharacterized protein (gene HMPREF0351_12038) | $4.90\times10^{-25}$ | Uncharacterized protein (gene HMPREF0351_12038) | yes |
| *E. faecalis* V583 | Uncharacterised protein (gene EF_0665) | $1.10\times10^{-39}$ | Uncharacterised protein (gene EF_0665) | yes |
| | 50S ribosomal protein L29 | $3.70\times10^{-39}$ | 50S ribosomal protein L29 | yes |
| | UPF0337 protein EF_1180 | $4.90\times10^{-48}$ | UPF0337 protein EF_1180 | yes |
| | DNA-binding protein HU | $1.40\times10^{-4}$ | DNA-binding protein HU | yes |
| *K. pneumoniae* KP257 | CscD domain-containing protein | N/A | - | no |
| | 30S ribosomal protein S16 | $6.50\times10^{-12}$ | 30S ribosomal protein S16 | yes |
| | DNA-binding protein HU-α | $7.63\times10^{-15}$ | DNA-binding protein HU-α | yes |
| | Uncharacterized protein (gene yciG) | $3.50\times10^{-8}$ | Uncharacterized protein (gene yciG) | yes |
| | 50S ribosomal protein L29 | $9.45\times10^{-8}$ | 50S ribosomal protein L29 | yes |
| | Uncharacterized protein KPN_00497 | $2.54\times10^{-18}$ | Uncharacterized protein KPN_00497 | yes |
| *A. baumannii* AYE | Uncharacterised protein (gene ABAYE1298) | $1.36\times10^{-6}$ | Uncharacterised protein (gene ABAYE1298) | yes |
| | Entericidin B | $1.70\times10^{-20}$ | Entericidin B | yes |
| | Uncharacterised protein (gene ABAYE2274) | $1.95\times10^{-11}$ | Uncharacterised protein (gene ABAYE2274) | yes |
| | Uncharacterised protein (gene ABAYE1876) | N/A | - | no |
| *E. cloacae* S11 | DNA-binding protein HU | $5.83\times10^{-57}$ | DNA-binding protein HU | yes |
| | CsbD family protein gene YjbJ | $6.20\times10^{-1}$ | CsbD family protein gene YjbJ | yes |
| | 50S ribosomal protein L29 | $2.10\times10^{-15}$ | 50S ribosomal protein L29 (*K. pneumoniae*) | no |
| | UPF0391 membrane protein SAMEA2054040_04753 | $1.69\times10^{-38}$ | UPF0391 membrane protein KPN_04833 (*K. pneumoniae*) | no |
| | DUF1471 domain-containing protein | $1.80\times10^{1}$ | Uncharacterized protein EF_2117 (*E. faecalis*) | no |

## Supplemental File 1: Protein assignments

**Protein ID assignments**

All masses listed are monoisotopic. Post-translational modifications: f – formylation.

***E. faecium* E745**

Protein name: Uncharacterized protein (gene HMPREF0351_11703, Uniprot accession no. Q3XZ91)
Charge state: +8
Observed monoisotopic mass: 5967.3058 Da
Sequence: fMKKFVSGILVGSLATAAAVAGLVASVKKTVIDPIDEKEAMIEENRKKAMRKRVSR

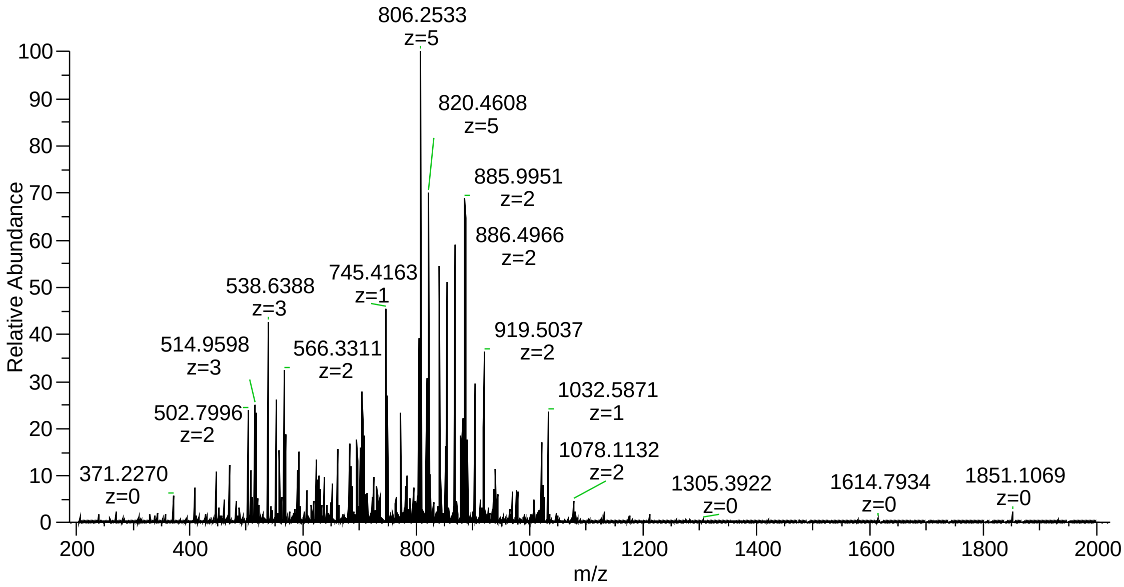


| Observed mass (monoisotopic) m/z | Theoretical mass (monoisotopic) m/z | Charge | Fragment | Mass error [Da] | Mass error [ppm] |
|---|---|---|---|---|---|
| 451.2499 | 451.2518 | 2 | b8-H2O | -0.0038 | -4.2105 |
| 460.2551 | 460.2571 | 2 | b8 | -0.0040 | -4.3454 |
| 502.7996 | 502.8007 | 2 | y8 | -0.0022 | -2.1877 |
| 507.7916 | 507.7939 | 2 | b9-H2O | -0.0046 | -4.5294 |
| 510.6454 | 510.6476 | 3 | y12 | -0.0066 | -4.3083 |
| 516.7971 | 516.7992 | 2 | b9 | -0.0042 | -4.0635 |
| 520.9632 | 520.9655 | 3 | b15 | -0.0069 | -4.4149 |
| 544.6422 | 544.6445 | 3 | b16 | -0.0069 | -4.2229 |
| 562.3174 | 562.32 | 3 | b17-H2O | -0.0078 | -4.6237 |
| 566.3311 | 566.3334 | 2 | b10 | -0.0046 | -4.0612 |
| 568.3211 | 568.3235 | 3 | b17 | -0.0072 | -4.2229 |

| | | | | | |
|---|---|---|---|---|---|
| 592.0002 | 592.0026 | 3 | b18 | -0.0072 | -4.0540 |
| 594.8416 | 594.8441 | 2 | b11 | -0.0050 | -4.2028 |
| 618.589 | 618.5916 | 4 | y20-H2O | -0.0104 | -4.2031 |
| 623.0917 | 623.0942 | 4 | Y20 | -0.0100 | -4.0122 |
| 625.0228 | 625.0254 | 3 | b19 | -0.0078 | -4.1598 |
| 638.3575 | 638.3601 | 2 | b12 | -0.0052 | -4.0729 |
| 648.7015 | 648.7044 | 3 | b20 | -0.0087 | -4.4704 |
| 662.3666 | 662.3694 | 1 | b5 | -0.0028 | -4.2272 |
| 683.5507 | 683.5535 | 6 | y36 | -0.0168 | -4.0962 |
| 694.8994 | 694.9021 | 2 | b13 | -0.0054 | -3.8854 |
| 700.0619 | 700.0649 | 6 | y37 | -0.0180 | -4.2853 |
| 703.6818 | 703.6817 | 7 | y46-NH3 | 0.0007 | 0.1421 |
| 704.3823 | 704.3852 | 4 | y23 | -0.0116 | -4.1171 |
| 711.9012 | 711.9044 | 6 | y38 | -0.0192 | -4.4950 |
| 723.7411 | 723.7439 | 6 | y39 | -0.0168 | -3.8688 |
| 730.4178 | 730.4207 | 2 | b14 | -0.0058 | -3.9703 |
| 735.5798 | 735.5834 | 6 | y40 | -0.0216 | -4.8941 |
| 761.2592 | 761.2625 | 6 | y42-H2O | -0.0198 | -4.3349 |
| 764.2616 | 764.2642 | 6 | y42 | -0.0156 | -3.4020 |
| 780.2734 | 780.2738 | 6 | y43-NH3 | -0.0024 | -0.5126 |
| 783.1093 | 783.1116 | 6 | y43 | -0.0138 | -2.9370 |
| 805.8521 | 805.8553 | 5 | y35 | -0.0160 | -3.9709 |
| 807.1172 | 807.1156 | 6 | b46 | 0.0096 | 1.9824 |
| 816.4599 | 816.4631 | 2 | b16 | -0.0064 | -3.9193 |
| 820.0597 | 820.0627 | 5 | y36 | -0.0150 | -3.6583 |
| 823.6296 | 823.6319 | 6 | y46 | -0.0138 | -2.7925 |
| 839.8731 | 839.8764 | 5 | y37 | -0.0165 | -3.9291 |
| 851.9783 | 851.9817 | 2 | b17 | -0.0068 | -3.9907 |
| 854.0803 | 854.0838 | 5 | y38 | -0.0175 | -4.0980 |
| 868.288 | 868.2913 | 5 | y39 | -0.0165 | -3.8006 |
| 882.4947 | 882.4987 | 5 | y40 | -0.0200 | -4.5326 |
| 902.7045 | 902.7082 | 5 | y41 | -0.0185 | -4.0988 |
| 913.5105 | 913.5103 | 5 | y42-NH3 | 0.0010 | 0.2189 |
| 916.9123 | 916.9156 | 5 | y42 | -0.0165 | -3.5990 |
| 937.0308 | 937.0344 | 2 | b19 | -0.0072 | -3.8419 |
| 938.8411 | 938.8445 | 3 | y23 | -0.0102 | -3.6215 |
| 968.3395 | 968.3372 | 5 | b46 | 0.0115 | 2.3752 |
| 1007.0637 | 1007.0673 | 4 | y35 | -0.0144 | -3.5747 |
| 1014.8777 | 1014.8815 | 3 | y25 | -0.0114 | -3.7443 |
| 1024.8225 | 1024.8266 | 4 | y36 | -0.0164 | -4.0007 |
| 1032.5871 | 1032.591 | 1 | b9 | -0.0039 | -3.7769 |

Protein name: Uncharacterized protein (gene HMPREF0351_11270, Uniprot accession no. I3U1K6)
Charge state: +6
Observed monoisotopic mass: 7294.8163 Da
Sequence:
fMNQEELFQKVKEMIKNGNFDGAKRFIEEHKEQLGPYKEKAQNLLKDVNIDSVKNKFKNLFK

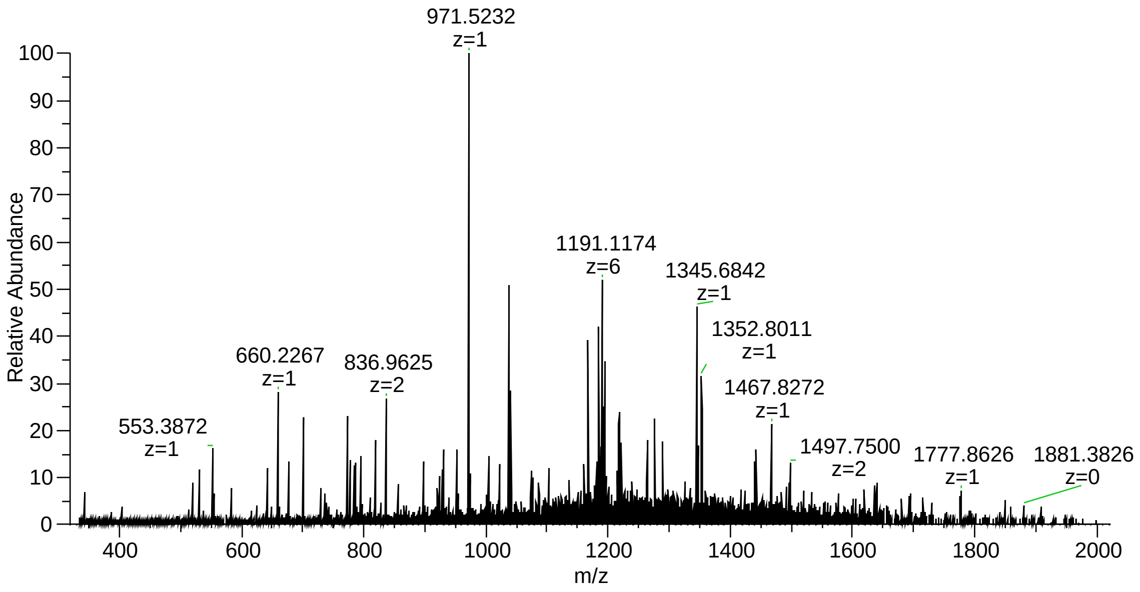


| Observed mass (monoisotopic) m/z | Theoretical mass (monoisotopic) m/z | Charge | Fragment | Mass error [Da] | Mass error [ppm] |
|---|---|---|---|---|---|
| 521.3059 | 521.3082 | 1 | y4 | -0.0023 | -4.4120 |
| 531.1846 | 531.1868 | 1 | b4 | -0.0022 | -4.1417 |
| 649.4005 | 649.4032 | 1 | y5 | -0.0027 | -4.1577 |
| 660.2268 | 660.2294 | 1 | b5 | -0.0026 | -3.9380 |
| 676.9035 | 676.9061 | 2 | y11 | -0.0052 | -3.8410 |
| 773.3107 | 773.3134 | 1 | b6 | -0.0027 | -3.4915 |
| 796.4685 | 796.4716 | 1 | y6 | -0.0031 | -3.8922 |
| 897.5134 | 897.5173 | 2 | y15 | -0.0078 | -4.3453 |
| 924.5628 | 924.5665 | 1 | y7 | -0.0037 | -4.0019 |
| 969.7331 | 969.7373 | 5 | y41 | -0.0210 | -4.3311 |
| 979.2926 | 979.2967 | 4 | y33 | -0.0164 | -4.1867 |
| 1010.0043 | 1010.0055 | 2 | b16 | -0.0024 | -1.1881 |
| 1019.074 | 1019.0782 | 2 | y17 | -0.0084 | -4.1214 |
| 1075.616 | 1075.6202 | 2 | y18 | -0.0084 | -3.9047 |
| 1093.6093 | 1093.6135 | 3 | y28 | -0.0126 | -3.8405 |

| | | | | | |
|---|---|---|---|---|---|
| 1095.533 | 1095.5377 | 2 | b18 | -0.0094 | -4.2901 |
| 1192.4541 | 1192.4574 | 6 | b60 | -0.0198 | -2.7674 |
| 1226.581 | 1226.5854 | 2 | b20 | -0.0088 | -3.5872 |
| 1275.5988 | 1275.6038 | 1 | b10 | -0.0050 | -3.9197 |
| 1288.7266 | 1288.7316 | 2 | y22 | -0.0100 | -3.8798 |
| 1352.801 | 1352.8049 | 1 | y11 | -0.0039 | -2.8829 |
| 1403.6938 | 1403.6988 | 1 | b11 | -0.0050 | -3.5620 |
| 1417.2952 | 1417.3004 | 2 | y24 | -0.0104 | -3.6689 |
| 1467.8265 | 1467.8318 | 1 | y12 | -0.0053 | -3.6108 |
| 1532.7355 | 1532.7414 | 1 | b12 | -0.0059 | -3.8493 |
| 1580.9105 | 1580.9159 | 1 | y13 | -0.0054 | -3.4157 |
| 1611.3997 | 1611.4059 | 2 | y27 | -0.0124 | -3.8476 |
| 1663.7762 | 1663.7818 | 1 | b13 | -0.0056 | -3.3658 |
| 1694.9527 | 1694.9588 | 1 | y14 | -0.0061 | -3.5989 |
| 1776.8605 | 1776.8659 | 1 | b14 | -0.0054 | -3.0391 |
| 1794.022 | 1794.0272 | 1 | y15 | -0.0052 | -2.8985 |
| 1909.0484 | 1909.0542 | 1 | y16 | -0.0058 | -3.0382 |

Protein name: 50S ribosomal protein L29 (Uniprot accession no. Q3XYY1)
Charge state: +8
Observed monoisotopic mass: 7339.0618 Da
Sequence: MKVKEIRELTTAEMLDQEKQLKEELFNLRFQLATGQLENTARIKEVRKSIARIKTVLREQAK

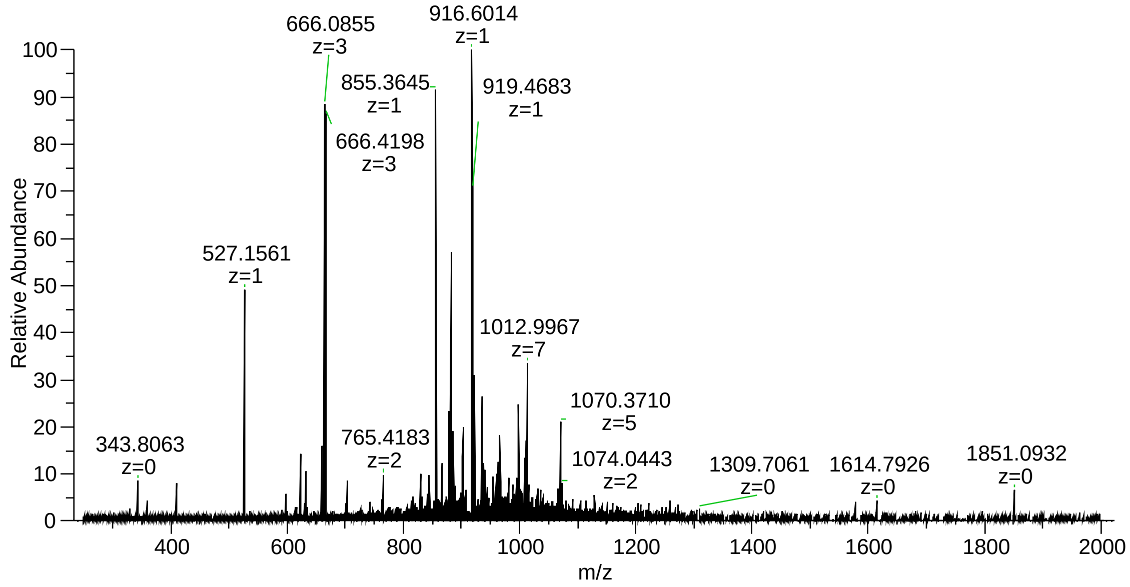

| Observed mass (monoisotopic) [Da] | Theoretical mass (monoisotopic) [Da] | Charge | Fragment | Mass error [Da] | Mass error [ppm] |
|---|---|---|---|---|---|
| 666.0855 | 666.0882 | 3 | y17 | -0.0081 | -4.0535 |
| 702.9274 | 702.9306 | 4 | y24 | -0.0128 | -4.5524 |
| 765.4183 | 765.4214 | 2 | b13 | -0.0062 | -4.0501 |
| 830.9382 | 830.9417 | 2 | b14 | -0.0070 | -4.2121 |
| 965.955 | 965.9586 | 5 | y41 | -0.0180 | -3.7269 |
| 1012.4237 | 1012.4272 | 7 | y60 | -0.0245 | -3.4570 |
| 1069.7749 | 1069.7737 | 5 | b45 | 0.0060 | 1.1217 |
| 1272.6768 | 1272.681 | 4 | b43 | -0.0168 | -3.3001 |

Protein name: 30S ribosomal protein S20 (Uniprot accession no. I3U1I4)
Charge state: +13
Observed monoisotopic mass: 9053.8009 Da
Sequence:
PNIESAIKRVRTSENANVKNSSQTSAMRTAIKKFEDAVASGADNVDALYKEAVKAIDMAESKGLIHKNKANRDKSRLSKKIAK

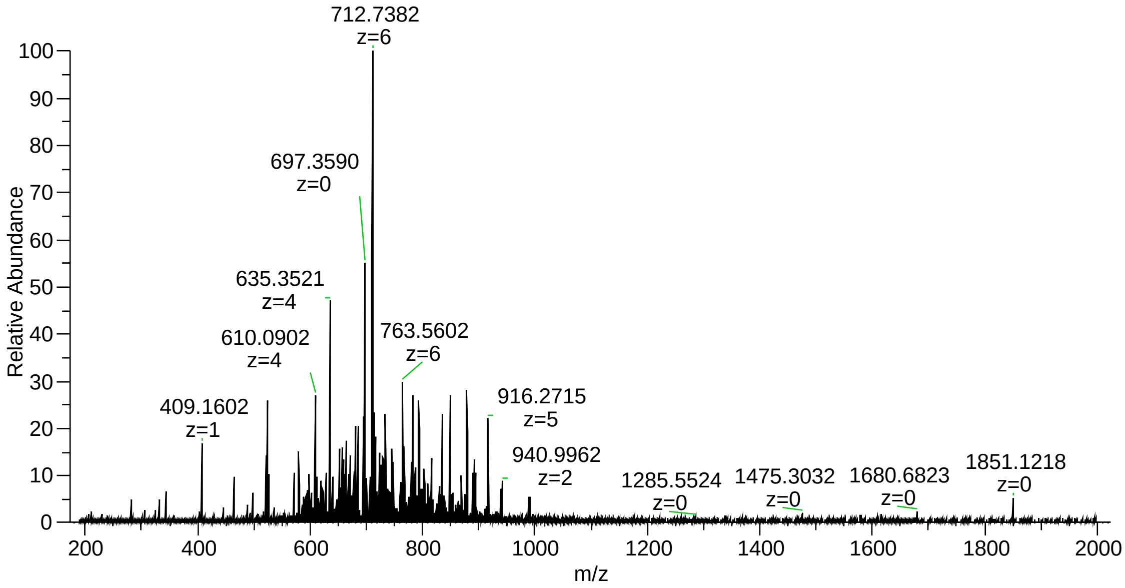


| Observed mass (monoisotopic) [Da] | Theoretical mass (monoisotopic) [Da] | Charge | Fragment | Mass error [Da] | Mass error [ppm] |
|---|---|---|---|---|---|
| 497.0555 | 497.0577 | 4 | y17 | -0.0088 | -4.4260 |
| 524.9204 | 524.9227 | 5 | y23 | -0.0115 | -4.3816 |
| 579.8851 | 579.8877 | 2 | y10 | -0.0052 | -4.4836 |
| 602.8264 | 602.8294 | 6 | b33 | -0.0180 | -4.9765 |

| | | | | | |
|---|---|---|---|---|---|
| 610.6323 | 610.6351 | 7 | y38 | -0.0196 | -4.5854 |
| 624.7848 | 624.7878 | 7 | y39 | -0.0210 | -4.8016 |
| 627.6666 | 627.6694 | 3 | b17 | -0.0084 | -4.4609 |
| 638.5041 | 638.5067 | 7 | y40-H2O | -0.0182 | -4.0720 |
| 641.0764 | 641.0796 | 7 | y40 | -0.0224 | -4.9916 |
| 654.3371 | 654.3402 | 7 | b43 | -0.0217 | -4.7376 |
| 655.8988 | 655.9016 | 4 | y23 | -0.0112 | -4.2689 |
| 662.3790 | 662.3818 | 6 | y35 | -0.0168 | -4.2272 |
| 668.0163 | 668.0190 | 6 | b36 | -0.0162 | -4.0418 |
| 670.6290 | 670.6321 | 7 | b44 | -0.0217 | -4.6225 |
| 681.2257 | 681.2291 | 6 | y36 | -0.0204 | -4.9910 |
| 684.7820 | 684.7847 | 7 | b45 | -0.0189 | -3.9428 |
| 708.2063 | 708.2095 | 6 | b39 | -0.0192 | -4.5184 |
| 709.2349 | 709.2380 | 6 | y38-H2O | -0.0186 | -4.3709 |
| 712.2369 | 712.2398 | 6 | y38 | -0.0174 | -4.0717 |
| 722.7116 | 722.7148 | 6 | b40 | -0.0192 | -4.4277 |
| 725.7465 | 725.7494 | 6 | y39-H2O | -0.0174 | -3.9959 |
| 728.7477 | 728.7512 | 6 | y39 | -0.0210 | -4.8027 |
| 747.7556 | 747.7583 | 6 | y40 | -0.0162 | -3.6108 |
| 760.3918 | 760.3913 | 6 | b43-NH3 | 0.0030 | 0.6576 |
| 775.9303 | 775.9313 | 6 | y42-NH3 | -0.0060 | -1.2888 |
| 782.2328 | 782.2362 | 6 | b44 | -0.0204 | -4.3465 |
| 788.2688 | 788.2726 | 6 | y43 | -0.0228 | -4.8207 |
| 835.4390 | 835.4425 | 5 | b38 | -0.0175 | -4.1894 |
| 849.6463 | 849.6499 | 5 | b39 | -0.0180 | -4.2370 |
| 867.0533 | 867.0563 | 5 | b40 | -0.0150 | -3.4600 |
| 878.4569 | 878.4606 | 5 | b41 | -0.0185 | -4.2119 |
| 892.6650 | 892.6680 | 5 | b42 | -0.0150 | -3.3607 |
| 915.6697 | 915.6734 | 5 | b43 | -0.0185 | -4.0407 |
| 938.4779 | 938.4820 | 5 | b44 | -0.0205 | -4.3688 |
| 940.9963 | 941.0005 | 2 | b17 | -0.0084 | -4.4633 |
| 990.5309 | 990.5347 | 2 | b18 | -0.0076 | -3.8363 |

Protein name: Uncharacterized protein (gene HMPREF0351_12038, Uniprot accession no. Q3XY98)
Charge state: +7
Observed monoisotopic mass: 6596.5391 Da
Sequence: MKWKEKVDEAAEKLYDLVKSEKYNIEVNIPKKGGKAVRVKSKRPTNHTKKWMAKNR

This protein contains an R->Q mutation at two possible positions 38 or 43.

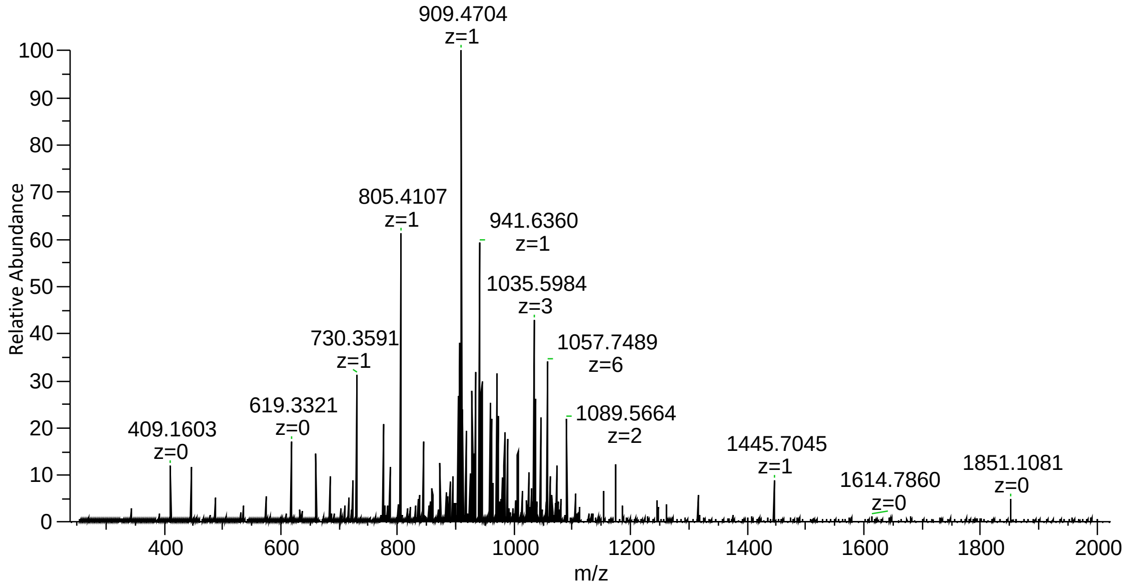


| Observed mass (monoisotopic) [Da] | Theoretical mass (monoisotopic) [Da] | Fragment | Mass error [Da] | Mass error [ppm] |
|---|---|---|---|---|
| 1044.5397 | 1044.5426 | B8 | -0.0030 | -2.8357 |
| 1173.5836 | 1173.5852 | B9 | -0.0016 | -1.3906 |
| 1444.6946 | 1444.7020 | B12 | -0.0074 | -5.1097 |
| 1444.6978 | 1444.7020 | B12 | -0.0042 | -2.9362 |
| 1572.7949 | 1572.7970 | B13 | -0.0021 | -1.3276 |
| 1685.8762 | 1685.8810 | B14 | -0.0049 | -2.8804 |
| 1963.9565 | 1963.9713 | B16 | -0.0148 | -7.5383 |
| 2176.117 | 2176.1238 | B18 | -0.0068 | -3.1207 |
| 804.4053 | 804.4065 | Y6 | -0.0012 | -1.4719 |
| 2749.5197 | 2777.5823 | Y24 | 0.0400 | 14.3945 |
| 3102.7611 | 3130.8250 | Y27 | 0.0387 | 12.3514 |
| 3102.7655 | 3130.8250 | Y27 | 0.0431 | 13.7523 |
| 3428.9627 | 3457.0204 | Y30 | 0.0449 | 12.9768 |
| 3558.0035 | 3586.0630 | Y31 | 0.0430 | 12.0026 |
| 3948.1766 | 3976.2533 | Y34 | 0.0258 | 6.5001 |
| 4292.3505 | 4320.4229 | Y37 | 0.0302 | 6.9905 |
| 4292.3516 | 4320.4229 | Y37 | 0.0313 | 7.2393 |
| 4420.4481 | 4448.5179 | Y38 | 0.0328 | 7.3777 |
| 4632.5809 | 4660.6703 | Y40 | 0.0131 | 2.8198 |
| 4910.6742 | 4938.7606 | Y42 | 0.0162 | 3.2781 |
| 5422.9968 | 5451.0564 | Y47 | 0.0430 | 7.8851 |
| 6337.4108 | 6365.5062 | Y54 | 0.0072 | 1.1272 |

***E. faecalis* V583**

Protein name: Uncharacterized protein (gene EF_0665, Uniprot accession no. Q838A7)
Charge state: +6
Observed monoisotopic mass: 6666.4375 Da
Sequence: MLSKEEVLHLLNEAKKEVDRLETNRQEDLGNSINYIENELQLQRVLSQVEAYEKVLG

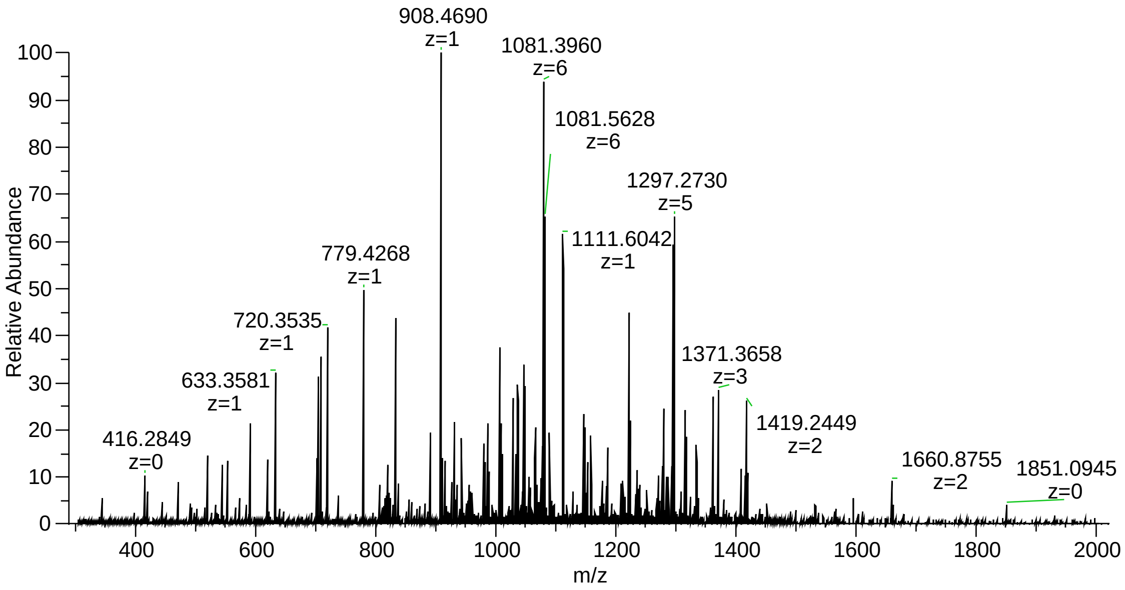


| Observed mass (monoisotopic) [Da] | Theoretical mass (monoisotopic) [Da] | Charge | Fragment | Mass difference [Da] | Mass difference [ppm] |
|---|---|---|---|---|---|
| 534.2792 | 534.2813 | 2 | b9 | -0.0042 | -2.3396 |
| 545.3271 | 545.3293 | 1 | y5 | -0.0022 | -4.5844 |
| 708.3899 | 708.3927 | 1 | y6 | -0.0028 | -3.5291 |
| 779.4268 | 779.4298 | 1 | y7 | -0.0030 | -3.2075 |
| 837.9329 | 837.9359 | 4 | b28 | -0.0120 | -0.7459 |
| 908.4690 | 908.4724 | 1 | y8 | -0.0034 | -2.7519 |
| 958.9978 | 959.0020 | 4 | b33 | -0.0168 | -0.6517 |
| 980.5431 | 980.5467 | 2 | y17 | -0.0072 | -1.2748 |
| 987.5086 | 987.5127 | 4 | b34 | -0.0164 | -0.6329 |
| 1007.5375 | 1007.5408 | 1 | y9 | -0.0033 | -2.4813 |
| 1028.2739 | 1028.2786 | 4 | b35 | -0.0188 | -0.6078 |
| 1037.0851 | 1037.0888 | 2 | y18 | -0.0074 | -1.2053 |
| 1047.5396 | 1047.5440 | 5 | b44 | -0.0220 | -0.4773 |

| | | | | | |
|---|---|---|---|---|---|
| 1056.5448 | 1056.5496 | 4 | b36 | -0.0192 | -0.5915 |
| 1064.2175 | 1064.2199 | 6 | b54 | -0.0144 | -0.3915 |
| 1067.5527 | 1067.5554 | 1 | b9 | -0.0027 | -2.3418 |
| 1080.7272 | 1080.7313 | 6 | b55 | -0.0246 | -0.3855 |
| 1088.8067 | 1088.8102 | 4 | b37 | -0.0140 | -0.5740 |
| 1149.5765 | 1149.5816 | 4 | b39 | -0.0204 | -0.5437 |
| 1152.8023 | 1152.8063 | 5 | b49 | -0.0200 | -0.4337 |
| 1158.6272 | 1158.6315 | 2 | y20 | -0.0086 | -1.0789 |
| 1209.8627 | 1209.8673 | 4 | b41 | -0.0184 | -0.5166 |
| 1223.1485 | 1223.1528 | 2 | y21 | -0.0086 | -1.0219 |
| 1240.6361 | 1240.6389 | 3 | b32 | -0.0084 | -0.6717 |
| 1251.2394 | 1251.2434 | 5 | b53 | -0.0200 | -0.3996 |
| 1270.1511 | 1270.1529 | 4 | b43 | -0.0072 | -0.4921 |
| 1285.4702 | 1285.4744 | 5 | y55 | -0.0210 | -0.3890 |
| 1296.6714 | 1296.6761 | 5 | b55 | -0.0235 | -0.3856 |
| 1309.1732 | 1309.1782 | 4 | b44 | -0.0200 | -0.4774 |
| 1316.3424 | 1316.3479 | 3 | b34 | -0.0165 | -0.6331 |
| 1333.9399 | 1333.9453 | 4 | b45 | -0.0216 | -0.4685 |
| 1362.2160 | 1362.2163 | 4 | b46 | -0.0012 | -0.4588 |
| 1370.6972 | 1370.7023 | 3 | b35 | -0.0153 | -0.6080 |
| 1408.3870 | 1408.3970 | 3 | b36 | -0.0300 | -0.5917 |
| 1418.2423 | 1418.2480 | 2 | y24 | -0.0114 | -0.8814 |
| 1440.7527 | 1440.7561 | 4 | b49 | -0.0136 | -0.4338 |
| 1451.4045 | 1451.4112 | 3 | b37 | -0.0201 | -0.5742 |
| 1532.4346 | 1532.4397 | 3 | b39 | -0.0153 | -0.5438 |
| 1660.3752 | 1660.3803 | 2 | y29 | -0.0102 | -0.7528 |

Protein name: 50S ribosomal protein L29 (Uniprot accession no. Q839F6)
Charge state: +7
Observed monoisotopic mass: 7324.9912 Da
Sequence: MKVKEIRELTTAEMLDKEKQLKEELFNLRFQLATGQLENTARIKEVRQSIARIKTVLREQAN

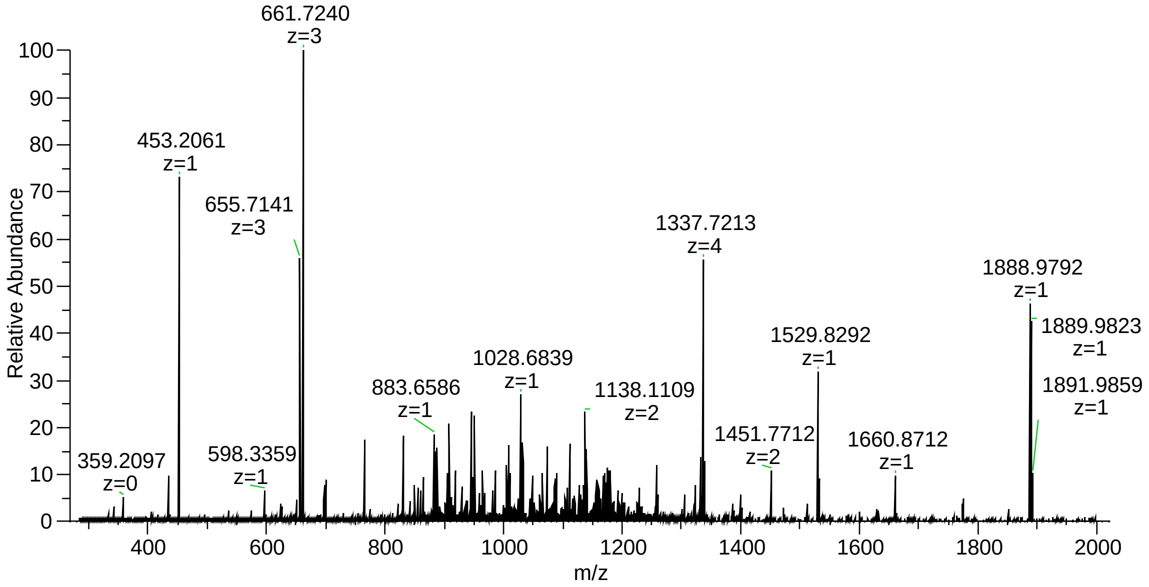


| Observed mass (monoisotopic) [Da] | Theoretical mass (monoisotopic) [Da] | Charge | Fragment | Mass difference [Da] | Mass difference [ppm] |
|---|---|---|---|---|---|
| 598.3359 | 598.3381 | 1 | b5-H2O | -0.0022 | -3.6769 |
| 655.7141 | 655.7166 | 3 | y17-H2O | -0.0075 | -3.8126 |
| 661.3897 | 661.3921 | 3 | y17 | -0.0072 | -3.6287 |
| 694.9029 | 694.9059 | 4 | y24-H2O | -0.0120 | -4.3171 |
| 699.4061 | 699.4085 | 4 | y24 | -0.0096 | -3.4315 |
| 765.4185 | 765.4214 | 2 | b13 | -0.0058 | -3.7888 |
| 830.9385 | 830.9417 | 2 | b14 | -0.0064 | -3.8511 |
| 855.4834 | 855.4865 | 2 | y15-NH3 | -0.0062 | -3.6237 |
| 863.9964 | 863.9998 | 2 | y15 | -0.0068 | -3.9352 |
| 881.8206 | 881.8239 | 3 | b22 | -0.0099 | -3.7422 |
| 885.9013 | 885.9049 | 5 | y38 | -0.0180 | -4.0636 |
| 887.4806 | 887.4837 | 2 | b15 | -0.0062 | -3.4930 |
| 904.3410 | 904.3434 | 6 | y46-H2O | -0.0144 | -2.6539 |
| 907.1779 | 907.1812 | 6 | y46 | -0.0198 | -3.6376 |
| 934.1179 | 934.1166 | 5 | y40-NH3 | 0.0065 | 1.3917 |
| 944.9937 | 944.9972 | 2 | b16 | -0.0070 | -3.7037 |
| 959.5366 | 959.5388 | 5 | y41-H2O | -0.0110 | -2.2928 |

| | | | | | |
|---|---|---|---|---|---|
| 963.1376 | 963.1409 | 5 | y41 | -0.0165 | -3.4263 |
| 967.0366 | 967.0398 | 6 | y49 | -0.0192 | -3.3091 |
| 982.3481 | 982.3524 | 5 | y42-NH3 | -0.0215 | -4.3772 |
| 985.7552 | 985.7577 | 5 | y42 | -0.0125 | -2.5361 |
| 1007.9634 | 1007.9641 | 5 | y43-NH3 | -0.0035 | -0.6945 |
| 1011.3688 | 1011.3694 | 5 | y43 | -0.0030 | -0.5933 |
| 1028.7150 | 1028.7191 | 7 | y61 | -0.0287 | -3.9855 |
| 1033.5798 | 1033.5831 | 5 | y44-NH3 | -0.0165 | -3.1928 |
| 1059.3920 | 1059.3916 | 5 | y45-NH3 | 0.0020 | 0.3776 |
| 1062.7936 | 1062.7970 | 5 | y45 | -0.0170 | -3.1991 |
| 1064.5569 | 1064.5607 | 2 | b18-H2O | -0.0076 | -3.5695 |
| 1073.5620 | 1073.5660 | 2 | b18 | -0.0080 | -3.7259 |
| 1085.0062 | 1085.0106 | 5 | y46-NH3 | -0.0220 | -4.0553 |
| 1108.0146 | 1108.0160 | 5 | y47-NH3 | -0.0070 | -1.2635 |
| 1111.4173 | 1111.4213 | 5 | y47 | -0.0200 | -3.5990 |
| 1116.4467 | 1116.4498 | 6 | y57-NH3 | -0.0186 | -2.7767 |
| 1137.6093 | 1137.6134 | 2 | b19 | -0.0082 | -3.6040 |
| 1162.1416 | 1162.1438 | 6 | y59 | -0.0132 | -1.8931 |
| 1167.3923 | 1167.3940 | 4 | y40-NH3 | -0.0068 | -1.4562 |
| 1171.6751 | 1171.6793 | 1 | y10 | -0.0042 | -3.5846 |
| 1175.6552 | 1175.6535 | 6 | y60-H2O | 0.0102 | 1.4460 |
| 1178.6494 | 1178.6552 | 6 | y60 | -0.0348 | -4.9209 |
| 1186.4511 | 1186.4499 | 5 | b50 | 0.0060 | 1.0114 |
| 1192.6332 | 1192.6374 | 2 | b20-H2O | -0.0084 | -3.5216 |
| 1197.1638 | 1197.1666 | 6 | y61-NH3 | -0.0168 | -2.3389 |
| 1199.4134 | 1199.4177 | 4 | y41-NH3 | -0.0172 | -3.5851 |
| 1227.6865 | 1227.6887 | 4 | y42-NH3 | -0.0088 | -1.7920 |
| 1258.1807 | 1258.1848 | 2 | b21 | -0.0082 | -3.2587 |
| 1300.2067 | 1300.2112 | 4 | b44-H2O | -0.0180 | -3.4610 |
| 1304.7080 | 1304.7138 | 4 | B44 | -0.0232 | -4.4454 |
| 1322.2279 | 1322.2322 | 2 | b22 | -0.0086 | -3.2521 |
| 1332.7188 | 1332.7178 | 4 | b45-NH3 | 0.0040 | 0.7503 |
| 1336.9692 | 1336.9744 | 4 | b45 | -0.0208 | -3.8894 |
| 1386.7483 | 1386.7535 | 2 | b23 | -0.0104 | -3.7498 |
| 1400.7882 | 1400.7930 | 1 | b12 | -0.0048 | -3.4266 |
| 1451.2686 | 1451.2748 | 2 | b24 | -0.0124 | -4.2721 |
| 1511.4811 | 1511.4800 | 3 | b38 | 0.0033 | 0.7278 |
| 1529.8292 | 1529.8356 | 1 | b13 | -0.0064 | -4.1835 |
| 1660.8712 | 1660.8761 | 1 | b14 | -0.0049 | -2.9503 |
| 1773.9542 | 1773.9601 | 1 | b15 | -0.0059 | -3.3259 |
| 1888.9792 | 1888.9871 | 1 | b16 | -0.0079 | -4.1821 |

Protein name: UPF0337 protein (gene EF_1180, Uniprot accession no. Q836D5)
Charge state: +11
Observed monoisotopic mass: 8873.5100 Da
Sequence:
ADLKGRFDDAKDKVEGTAKEAQGKVTDDKGKELEGKAQSTFADVKDKARDAGDDLKEGAEKLTDKVKE
GFEDLKDKFSKDK

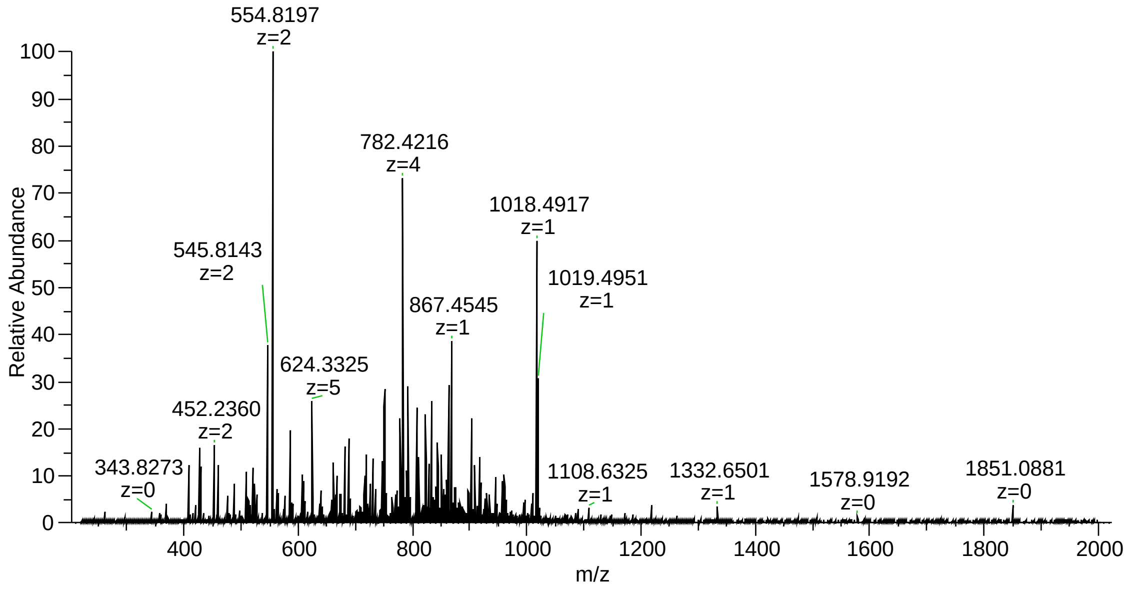


| Observed mass (monoisotopic) [Da] | Theoretical mass (monoisotopic) [Da] | Charge | Fragment | Mass difference [Da] | Mass difference [ppm] |
|---|---|---|---|---|---|
| 428.2122 | 428.2504 | 1 | b4 | -0.0382 | -5.8377 |
| 452.2360 | 452.2378 | 2 | b8 | -0.0036 | -2.7640 |
| 487.5859 | 487.5879 | 3 | b13 | -0.0060 | -1.7091 |
| 498.2776 | 498.2796 | 2 | y8 | -0.0040 | -2.5086 |
| 509.7492 | 509.7513 | 2 | b9 | -0.0042 | -2.4522 |
| 520.6087 | 520.6107 | 3 | b14 | -0.0060 | -1.6007 |
| 554.8197 | 554.8217 | 2 | y9 | -0.0040 | -2.2530 |
| 563.6228 | 563.6249 | 3 | b15 | -0.0063 | -1.4785 |
| 624.3325 | 624.3352 | 1 | y5 | -0.0027 | -4.0043 |
| 640.5819 | 640.5845 | 4 | b24 | -0.0104 | -0.9757 |
| 661.3426 | 661.3453 | 5 | b31 | -0.0135 | -0.7560 |
| 665.3484 | 665.3516 | 4 | b25 | -0.0128 | -0.9394 |
| 666.8283 | 666.8308 | 2 | b12 | -0.0050 | -1.8745 |

| | | | | | |
|---|---|---|---|---|---|
| 716.8758 | 716.8787 | 6 | y38 | -0.0174 | -0.5812 |
| 719.3672 | 719.3703 | 4 | b27 | -0.0124 | -0.8688 |
| 721.8750 | 721.8789 | 4 | y25 | -0.0156 | -0.8658 |
| 730.8752 | 730.8782 | 2 | b13 | -0.0060 | -1.7103 |
| 748.1232 | 748.1270 | 4 | b28 | -0.0152 | -0.8354 |
| 782.1707 | 782.1737 | 4 | y27 | -0.0120 | -0.7991 |
| 790.7713 | 790.7753 | 11 | y79 | -0.0440 | -0.2874 |
| 791.6122 | 791.6149 | 5 | y35 | -0.0135 | -0.6316 |
| 822.2677 | 822.2711 | 7 | b54 | -0.0238 | -0.4343 |
| 841.7242 | 841.7263 | 7 | y53 | -0.0147 | -0.4243 |
| 843.4195 | 843.4227 | 2 | y14 | -0.0064 | -1.4821 |
| 855.1819 | 855.1852 | 8 | b64 | -0.0264 | -0.3654 |
| 858.1564 | 858.1588 | 7 | y54 | -0.0168 | -0.4162 |
| 863.8798 | 863.8832 | 9 | b72 | -0.0306 | -0.3215 |
| 867.4545 | 867.4571 | 1 | y7 | -0.0026 | -2.8820 |
| 873.7424 | 873.7480 | 10 | b80 | -0.0560 | -0.2861 |
| 903.4649 | 903.4683 | 1 | b8 | -0.0034 | -2.7671 |
| 907.4660 | 907.4702 | 2 | y15 | -0.0084 | -1.3775 |
| 916.6606 | 916.6640 | 5 | b43 | -0.0170 | -0.5455 |
| 919.1513 | 919.1553 | 3 | y24 | -0.0120 | -0.9066 |
| 929.6402 | 929.6443 | 6 | y50 | -0.0246 | -0.4482 |
| 959.1551 | 959.1484 | 6 | b54 | 0.0402 | -0.4344 |
| 962.1661 | 962.1695 | 3 | y25 | -0.0102 | -0.8661 |
| 964.2927 | 964.2954 | 5 | y43 | -0.0135 | -0.5185 |
| 997.1636 | 997.1669 | 3 | b28 | -0.0099 | -0.8357 |
| 1018.4917 | 1018.4952 | 1 | b9 | -0.0035 | -2.4546 |
| 1108.6325 | 1108.6361 | 1 | y9 | -0.0036 | -2.2550 |
| 1217.6236 | 1217.6273 | 1 | b11 | -0.0037 | -2.0532 |
| 1332.6501 | 1332.6543 | 1 | b12 | -0.0042 | -1.8760 |

Protein name: DNA-binding protein HU (Uniprot accession no. Q834T3)
Charge state: +11
Observed monoisotopic mass: 9519.1340 Da
Sequence:
ANKAELIENVASSTGLTKKDATAAVDAVFSTIQETLAKGEKVQLIGFGNFEVRERAARKGRNPQTGQEIQI
AASKVPAFKPGKALKDAVK

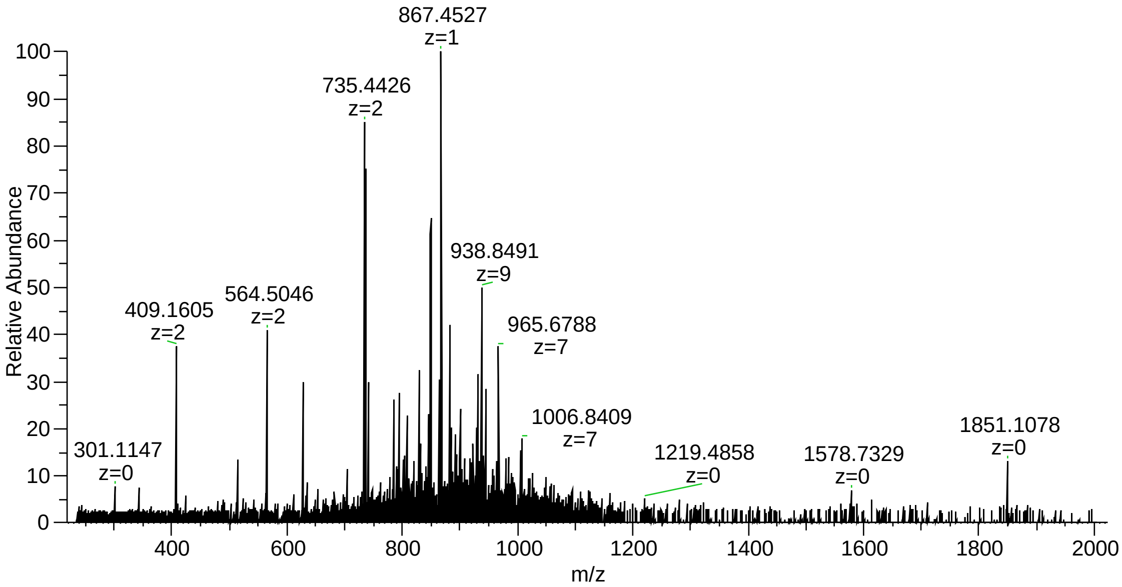


| Observed mass (monoisotopic) [Da] | Theoretical mass (monoisotopic) [Da] | Charge | Fragment | Mass difference [Da] | Mass difference [ppm] |
|---|---|---|---|---|---|
| 627.3436 | 627.3461 | 1 | b6 | -0.0025 | -3.9850 |
| 735.4427 | 735.4456 | 2 | y14 | -0.0058 | -1.6996 |
| 740.4271 | 740.4301 | 1 | b7 | -0.0030 | -3.3764 |
| 784.9765 | 784.9798 | 2 | y15 | -0.0066 | -1.5924 |
| 844.8431 | 844.8473 | 8 | y62 | -0.0336 | -0.3699 |
| 930.6203 | 930.6253 | 9 | y79 | -0.0450 | -0.2985 |
| 938.5102 | 938.5183 | 9 | y80 | -0.0729 | -0.2960 |
| 944.3824 | 944.3861 | 7 | y61 | -0.0259 | -0.3782 |
| 965.3935 | 965.3959 | 7 | y62 | -0.0168 | -0.3699 |
| 979.5453 | 979.5485 | 7 | y63 | -0.0224 | -0.3646 |
| 989.6929 | 989.6967 | 7 | y64 | -0.0266 | -0.3609 |
| 1006.1261 | 1006.1291 | 7 | y65 | -0.0210 | -0.3550 |
| 1020.2806 | 1020.2817 | 7 | y66 | -0.0077 | -0.3500 |
| 1082.5814 | 1082.5840 | 1 | b10 | -0.0026 | -2.3093 |

***K. pneumoniae* KP257**

Protein name: CscD domain-containing protein (Uniprot accession no. A6TGV4)
Charge state: +9
Observed monoisotopic mass: 8303.0402 Da
Sequence:
**MSGTKRAVNPPNDEDVI**MNKDEIGGNWKQFKGKAKEQWGKLTDDDMTVIEGKRDQLVGKIQERYG
YEKDQAEKEVSDWEHKNDYRW
Signal peptide 1-17 cleaved

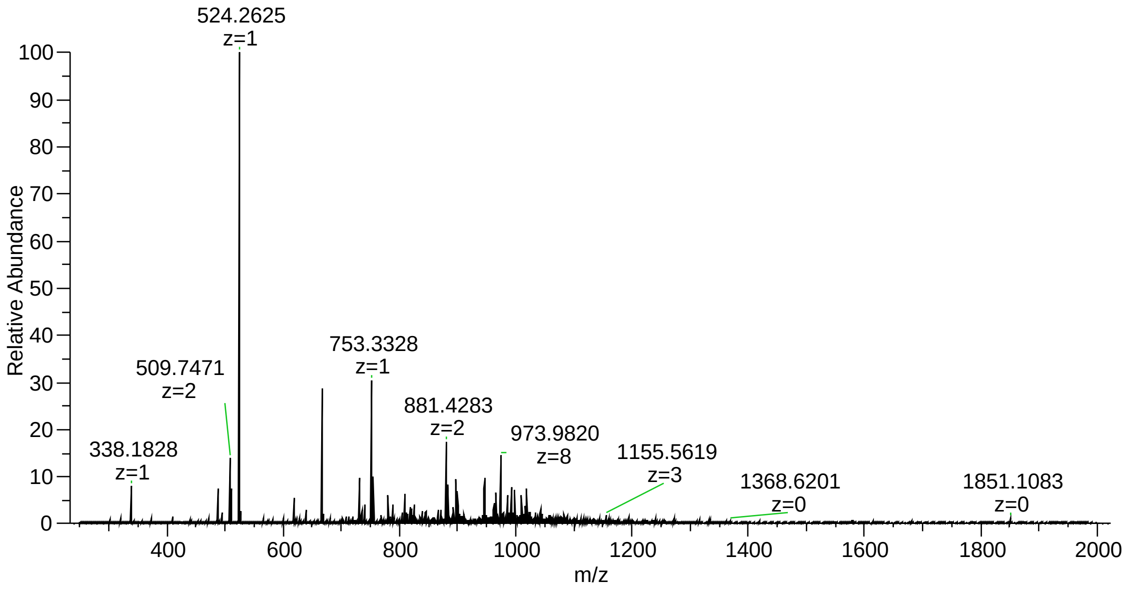


| Observed mass (monoisotopic) [Da] | Theoretical mass (monoisotopic) [Da] | Charge | Fragment | Mass difference [Da] | Mass difference [ppm] |
|---|---|---|---|---|---|
| 509.7471 | 509.7463 | 2 | y7 | 0.0016 | 1.5694 |
| 524.2625 | 524.2616 | 1 | y3 | 0.0009 | 1.7167 |
| 618.2560 | 618.2552 | 1 | b5 | 0.0008 | 1.2940 |
| 667.3085 | 667.3073 | 2 | y9 | 0.0024 | 1.7983 |
| 731.3404 | 731.3393 | 1 | b6 | 0.0011 | 1.5041 |
| 740.6763 | 740.6750 | 3 | y17 | 0.0039 | 1.7552 |
| 753.3328 | 753.3315 | 1 | y5 | 0.0013 | 1.7257 |
| 768.3382 | 768.3367 | 2 | y11 | 0.0030 | 1.9523 |
| 788.3922 | 788.3907 | 6 | y38 | 0.0090 | 1.9026 |
| 804.9036 | 804.9021 | 6 | y39 | 0.0090 | 1.8636 |
| 812.9020 | 812.9003 | 4 | b28 | 0.0068 | 2.0913 |
| 817.8726 | 817.8710 | 2 | y12 | 0.0032 | 1.9563 |
| 821.7437 | 821.7434 | 6 | y40 | 0.0018 | 0.3651 |

| | | | | | |
|---|---|---|---|---|---|
| 835.4242 | 835.4216 | 3 | b21 | 0.0078 | 3.1122 |
| 838.9157 | 838.9143 | 2 | b14 | 0.0028 | 1.6688 |
| 843.5844 | 843.5835 | 6 | y41 | 0.0054 | 1.0669 |
| 845.6619 | 845.6604 | 4 | b29 | 0.0060 | 1.7738 |
| 881.4283 | 881.4264 | 1 | y6 | 0.0019 | 2.1556 |
| 897.4438 | 897.4421 | 5 | y36 | 0.0085 | 1.8943 |
| 945.8691 | 945.8674 | 5 | y38 | 0.0085 | 1.7973 |
| 965.6823 | 965.6811 | 5 | y39 | 0.0060 | 1.2426 |
| 973.4801 | 973.4792 | 8 | b66 | 0.0072 | 0.9245 |
| 985.8920 | 985.8907 | 5 | y40 | 0.0065 | 1.3186 |
| 992.2394 | 992.2387 | 8 | y66 | 0.0056 | 0.7055 |
| 996.7831 | 996.7829 | 7 | b60 | 0.0014 | 0.2006 |
| 1008.2471 | 1008.2505 | 8 | y67 | -0.0272 | -3.3722 |
| 1015.0155 | 1015.0116 | 6 | b52 | 0.0234 | 3.8423 |
| 1018.4875 | 1018.4853 | 1 | y7 | 0.0022 | 2.1601 |

Protein name: 30S ribosomal protein S16 (Uniprot accession no. A6TCL7)
Charge state: +10
Observed monoisotopic mass: 9084.8622 Da
Sequence:
MVTIRLARHGAKKRPFYQVVVTDSRNARNGRFIERVGFFNPIANGAEEETRLDLDRIAHWVGQGATVSD
RVAALIKAANKAA

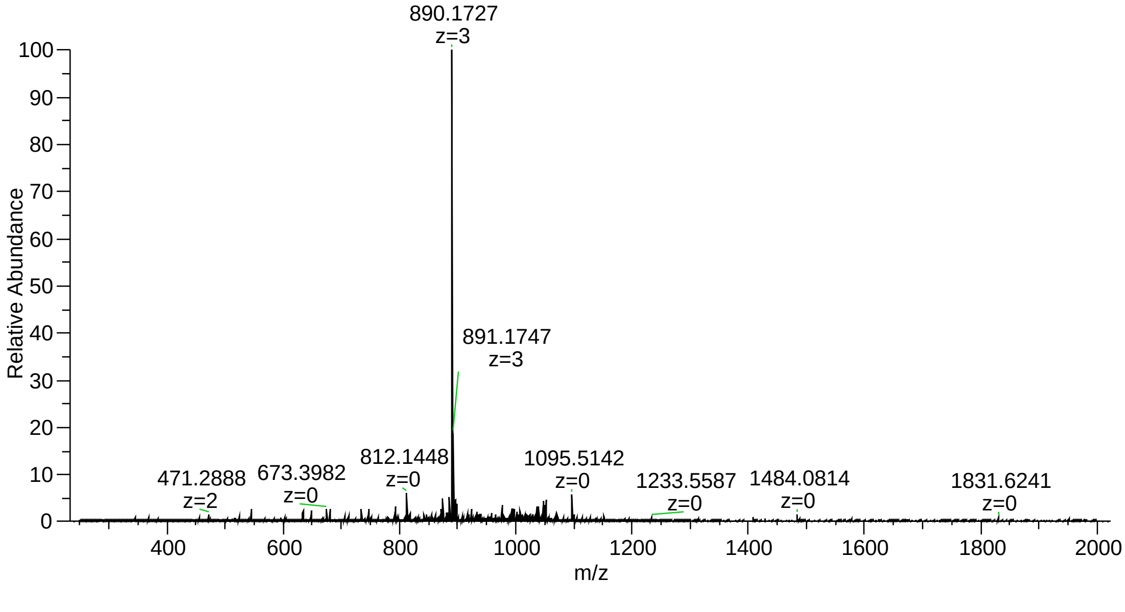

| Observed mass (monoisotopic) [Da] | Theoretical mass (monoisotopic) [Da] | Charge | Fragment | Mass difference [Da] | Mass difference [ppm] |
|---|---|---|---|---|---|
| 540.3307 | 540.3315 | 3 | b14 | -0.0024 | -1.4806 |
| 545.3036 | 545.3042 | 1 | y6 | -0.0006 | -1.1003 |
| 648.9084 | 648.9092 | 2 | y13 | -0.0016 | -1.2328 |
| 673.3986 | 673.3991 | 1 | y7 | -0.0005 | -0.7425 |
| 751.4208 | 751.4216 | 4 | y29 | -0.0032 | -1.0646 |
| 782.2627 | 782.2641 | 6 | b40 | -0.0084 | -1.7897 |
| 798.4390 | 798.4396 | 6 | b41 | -0.0036 | -0.7515 |
| 817.2847 | 817.2869 | 6 | b42 | -0.0132 | -2.6918 |
| 829.1270 | 829.1265 | 6 | b43 | 0.0030 | 0.6030 |
| 872.7359 | 872.7366 | 4 | y33 | -0.0028 | -0.8021 |
| 889.8383 | 889.8391 | 3 | b23 | -0.0024 | -0.8990 |
| 893.4819 | 893.4849 | 10 | b80 | -0.0300 | -3.3576 |
| 902.6218 | 902.6237 | 7 | b55 | -0.0133 | -2.1050 |
| 917.9172 | 917.9167 | 7 | y59 | 0.0035 | 0.5447 |
| 933.9986 | 933.9980 | 6 | b49 | 0.0036 | 0.6424 |
| 937.2569 | 937.2579 | 4 | y35 | -0.0040 | -1.0669 |
| 1014.8732 | 1014.8746 | 6 | b53 | -0.0084 | -1.3795 |
| 1019.0418 | 1019.0445 | 8 | y74 | -0.0216 | -2.6495 |
| 1024.2937 | 1024.2950 | 8 | b73 | -0.0104 | -1.2692 |
| 1030.0464 | 1030.0439 | 4 | y39 | 0.0100 | 2.4271 |
| 1038.4304 | 1038.4305 | 8 | b74 | -0.0008 | -0.0963 |
| 1047.8026 | 1047.8032 | 4 | y40 | -0.0024 | -0.5726 |
| 1052.5620 | 1052.5660 | 8 | b75 | -0.0320 | -3.8002 |
| 1100.3381 | 1100.3374 | 4 | y42 | 0.0028 | 0.6362 |
| 1334.2555 | 1334.2550 | 2 | b23 | 0.0010 | 0.3747 |

Protein name: Uncharacterized protein (gene yciG, Uniprot accession no. A6T7L1)
Charge state: +6
Observed monoisotopic mass: 6147.8875 Da
Sequence:
AEHRGGSGNFAEDREKASEAGRKGGQHSGGNFKNDPQRASEAGKKGGQNSHGGGRKSDNS

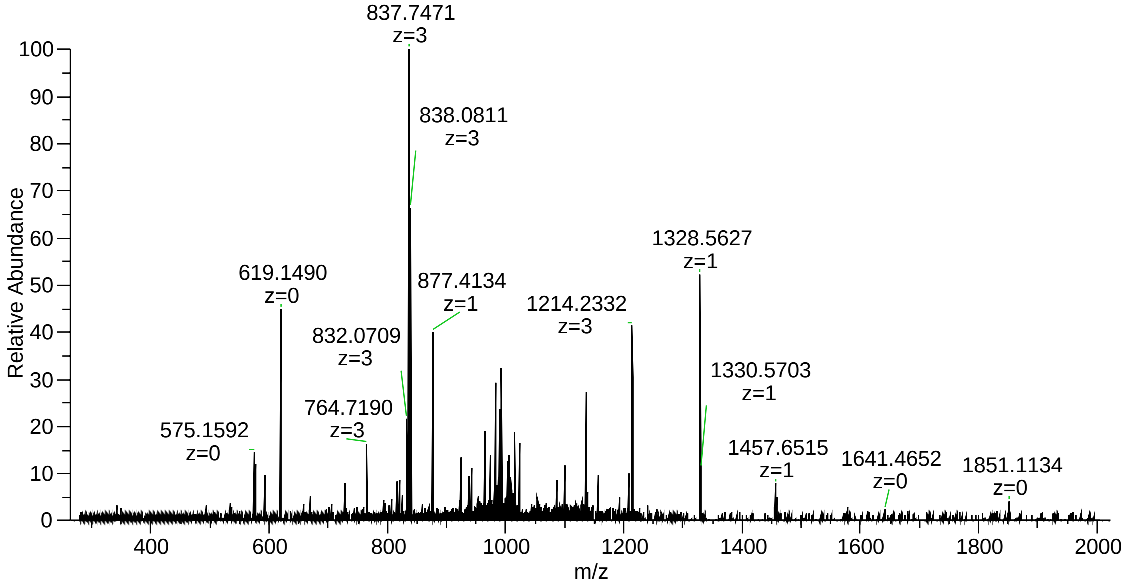


| Observed mass (monoisotopic) [Da] | Theoretical mass (monoisotopic) [Da] | Charge | Fragment | Mass difference [Da] | Mass difference [ppm] |
|---|---|---|---|---|---|
| 837.4128 | 837.4116 | 3 | y25 | 0.0036 | 1.4330 |
| 877.4134 | 877.4122 | 1 | y9 | 0.0012 | 1.3677 |
| 965.0732 | 965.0732 | 5 | y47 | 0.0000 | 0.0000 |
| 989.1400 | 989.1403 | 6 | b58 | -0.0018 | -0.3033 |
| 992.3099 | 992.3080 | 6 | y58 | 0.0114 | 1.9147 |
| 1213.5364 | 1213.5345 | 1 | b12 | 0.0019 | 1.5657 |
| 1328.5627 | 1328.5614 | 1 | b13 | 0.0013 | 0.9785 |
| 1457.6515 | 1457.6476 | 1 | y15 | 0.0039 | 2.6755 |

Protein name: 50S ribosomal protein L29 (gene rpmC, Uniprot accession no. A6TEW4)
Charge state: +7
Observed monoisotopic mass: 7239.0382 Da
Sequence:
MKAKELREKSVEELNAELLNLLREQFNLRMQAASGQLQQTHLLKQVRRDVARVKTLLTQKAGA

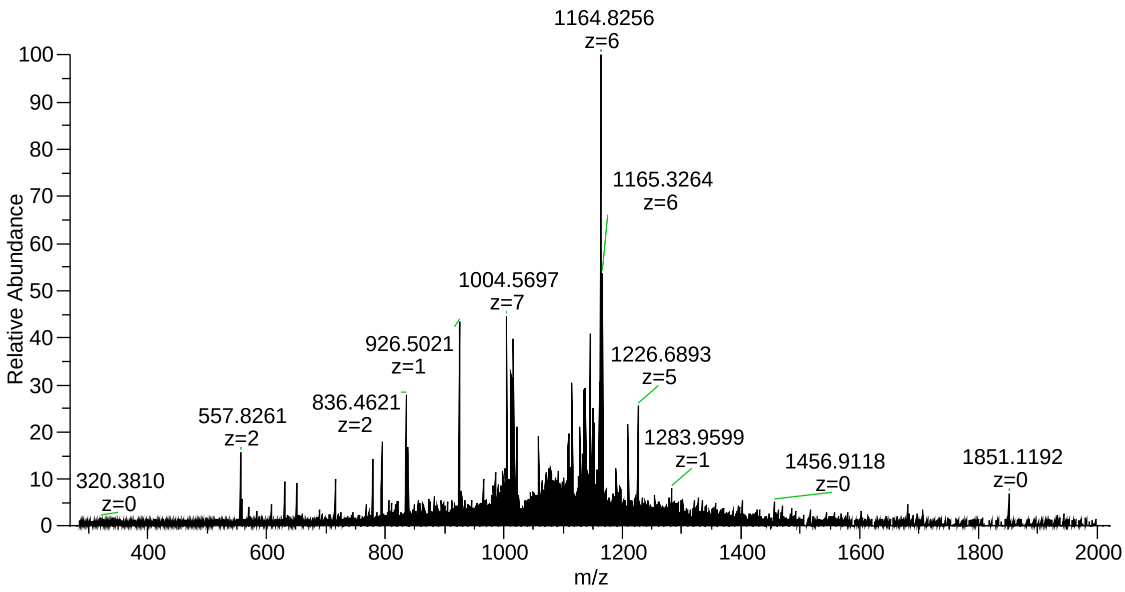


| Observed mass (monoisotopic) [Da] | Theoretical mass (monoisotopic) [Da] | Charge | Fragment | Mass difference [Da] | Mass difference [ppm] |
|---|---|---|---|---|---|
| 557.8260 | 557.8237 | 2 | b9 | 0.0046 | 4.1232 |
| 650.8765 | 650.8739 | 2 | b11 | 0.0052 | 3.9946 |
| 715.3985 | 715.3952 | 2 | b12 | 0.0066 | 4.6128 |
| 728.4562 | 728.4539 | 2 | y14 | 0.0046 | 3.1574 |
| 779.9196 | 779.9165 | 2 | b13 | 0.0062 | 3.9748 |
| 836.4620 | 836.4585 | 2 | b14 | 0.0070 | 4.1843 |
| 882.8727 | 882.8735 | 5 | b38 | -0.0040 | -0.9061 |
| 893.4833 | 893.4800 | 2 | b15 | 0.0066 | 3.6934 |
| 993.5233 | 993.5199 | 2 | b17 | 0.0068 | 3.4222 |
| 1004.1397 | 1004.1362 | 7 | b60 | 0.0245 | 3.4856 |
| 1014.2888 | 1014.2844 | 7 | b61 | 0.0308 | 4.3380 |
| 1077.6154 | 1077.6125 | 5 | y47 | 0.0145 | 2.6911 |
| 1109.6266 | 1109.6237 | 6 | y58 | 0.0174 | 2.6135 |
| 1152.4829 | 1152.4800 | 6 | y60 | 0.0174 | 2.5163 |
| 1163.0584 | 1163.0539 | 5 | y51 | 0.0225 | 3.8691 |
| 1164.3238 | 1164.3195 | 6 | y61 | 0.0258 | 3.6931 |
| 1208.6802 | 1208.6761 | 5 | y53 | 0.0205 | 3.3921 |

| | | | | | |
|---|---|---|---|---|---|
| 1226.0877 | 1226.0825 | 5 | y54 | 0.0260 | 4.2412 |
| 1455.9068 | 1455.9006 | 1 | y14 | 0.0062 | 4.2585 |

### *A. baumannii* AYE

Protein name: Uncharacterized protein (gene ABAYE1298, Uniprot accession no. B0V801)
Charge state: +9
Observed monoisotopic mass: 8713.3637 Da
Sequence:
MNTLNINDIKKHADVIASCGTKVGTVDHLEGENQLKLTKDENDQHHLIPTSWIGEVKEDQVILNKNSEEV
KENWQAI

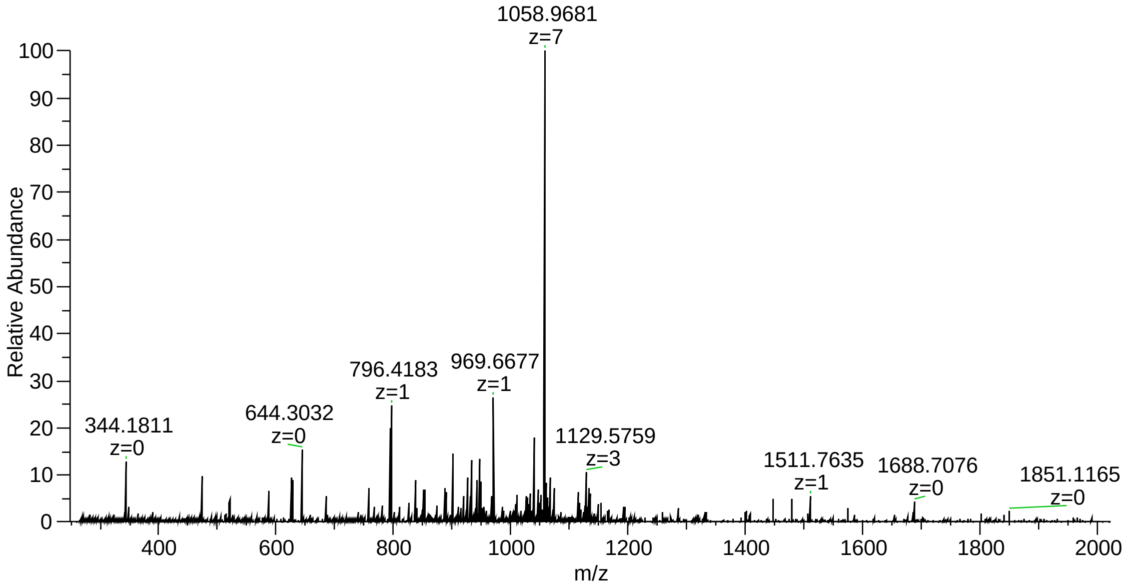


| Observed mass (monoisotopic) [Da] | Theoretical mass (monoisotopic) [Da] | Charge | Fragment | Mass difference [Da] | Mass difference [ppm] |
|---|---|---|---|---|---|
| 888.4559 | 888.4574 | 1 | y7 | -0.0015 | -1.6883 |
| 889.4547 | 889.4535 | 6 | b48 | 0.0072 | 1.3491 |
| 941.4726 | 941.4739 | 5 | b43 | -0.0065 | -1.3808 |
| 1032.7765 | 1032.7781 | 8 | y73 | -0.0128 | -1.5492 |
| 1073.7964 | 1073.8000 | 8 | y76 | -0.0288 | -3.3526 |
| 1046.9146 | 1046.9137 | 8 | y74 | 0.0072 | 0.8597 |
| 1125.9684 | 1125.9677 | 5 | y48 | 0.0035 | 0.6217 |
| 1128.5739 | 1128.5754 | 3 | y29 | -0.0045 | -1.3291 |
| 1134.2387 | 1134.2402 | 6 | b61 | -0.0090 | -1.3225 |
| 1153.0863 | 1153.0875 | 6 | b62 | -0.0072 | -1.0407 |

| | | | | | |
|---|---|---|---|---|---|
| 1166.2678 | 1166.2701 | 3 | y30 | -0.0069 | -1.9721 |
| 1285.6933 | 1285.6933 | 1 | b11 | 0.0000 | 0.0000 |
| 1332.6435 | 1332.6430 | 1 | y11 | 0.0005 | 0.3752 |
| 1446.6844 | 1446.6859 | 1 | y12 | -0.0015 | -1.0369 |

Protein name: Uncharacterized protein (gene ABAYE2274, Uniprot accession no. B0VBH8)
Charge state: +12
Observed monoisotopic mass: 12309.7539 Da
Sequence:
TDQNTDHAQLVAGDHNYSDSRWRESYLTRPYYQEAQLTTPDLDYDRDFSAAYELGHRARSESKEGTQF
KDMEGSLQQKWEELKAESRLKWEHAKQAIKDAWDDM

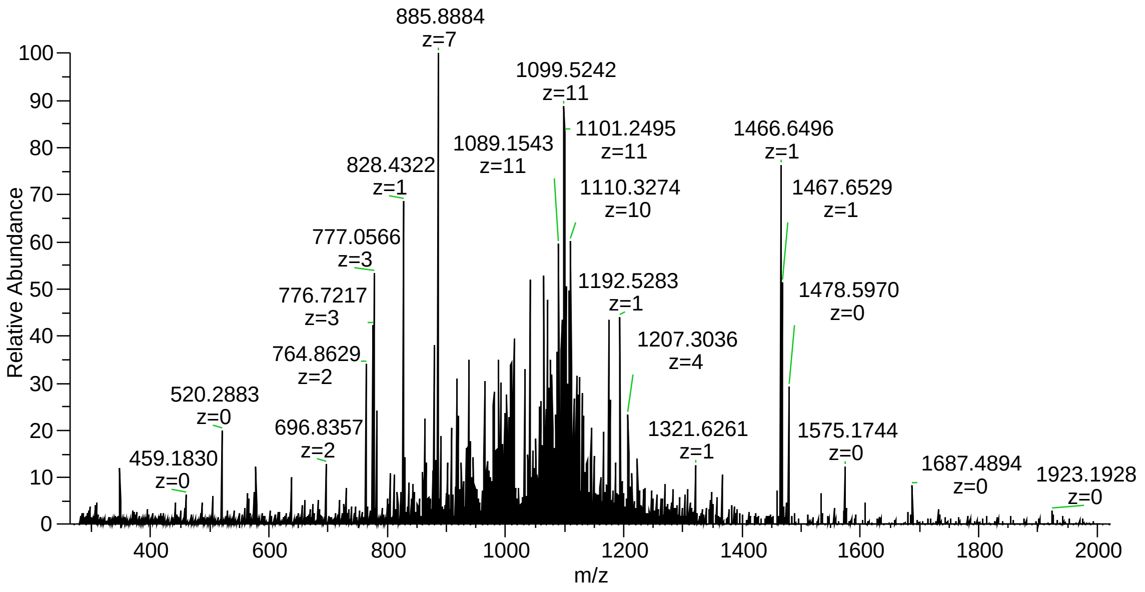


| Fragment | Observed mass (monoisotopic) [Da] | Theoretical mass (monoisotopic) [Da] | Mass difference [Da] | Mass difference [ppm] |
|---|---|---|---|---|
| B7 | 811.3106 | 811.3097 | 0.0009 | 1.1660 |
| B10 | 1123.4891 | 1123.4894 | -0.0003 | -0.2563 |
| B14 | 1465.6493 | 1465.6433 | 0.0059 | 4.0515 |
| B19 | 2081.8510 | 2081.8675 | -0.0165 | -7.9140 |
| B36 | 4295.9052 | 4295.9281 | -0.0229 | -5.3381 |
| B41 | 4823.1853 | 4823.1872 | -0.0019 | -0.3948 |
| B72 | 8455.7853 | 8455.8462 | -0.0609 | -7.1995 |
| B78 | 9097.1577 | 9097.1959 | -0.0382 | -4.2010 |
| Y7 | 879.3412 | 879.3433 | -0.0021 | -2.3483 |

| | | | | |
|---|---|---|---|---|
| Y8 | 992.4244 | 992.4273 | -0.0029 | -2.9554 |
| Y9 | 1063.4650 | 1063.4644 | 0.0006 | 0.5473 |
| Y10 | 1191.5166 | 1191.5230 | -0.0064 | -5.3654 |
| Y11 | 1319.6129 | 1319.6180 | -0.0051 | -3.8579 |
| Y13 | 1527.7159 | 1527.7140 | 0.0019 | 1.2745 |
| Y19 | 2327.1421 | 2327.1480 | -0.0060 | -2.5740 |
| Y23 | 2768.3935 | 2768.4068 | -0.0133 | -4.8002 |
| Y35 | 4229.0299 | 4229.0309 | -0.0010 | -0.2469 |
| Y51 | 6040.9574 | 6040.9700 | -0.0126 | -2.0914 |
| Y63 | 7486.5120 | 7486.5799 | -0.0679 | -9.0677 |
| Y63 | 7486.5346 | 7486.5799 | -0.0453 | -6.0523 |
| Y65 | 7698.6510 | 7698.6596 | -0.0086 | -1.1193 |
| Y92 | 11016.1012 | 11016.1722 | -0.0710 | -6.4468 |
| Y93 | 11087.2128 | 11087.2093 | 0.0035 | 0.3134 |
| Y94 | 11186.1847 | 11186.2777 | -0.0930 | -8.3164 |
| Y102 | 12093.6962 | 12093.6925 | 0.0037 | 0.3088 |

Protein name: Uncharacterized protein (gene ABAYE1876, Uniprot accession no. B0V4B5)
Charge state: +6
Observed monoisotopic mass: 6323.1751 Da
Sequence: MLKSILVLGTGIAIGMCMYKKKQKNSKSFSTDSDTDSLISKDNNKSKDDNSLDNAVQV

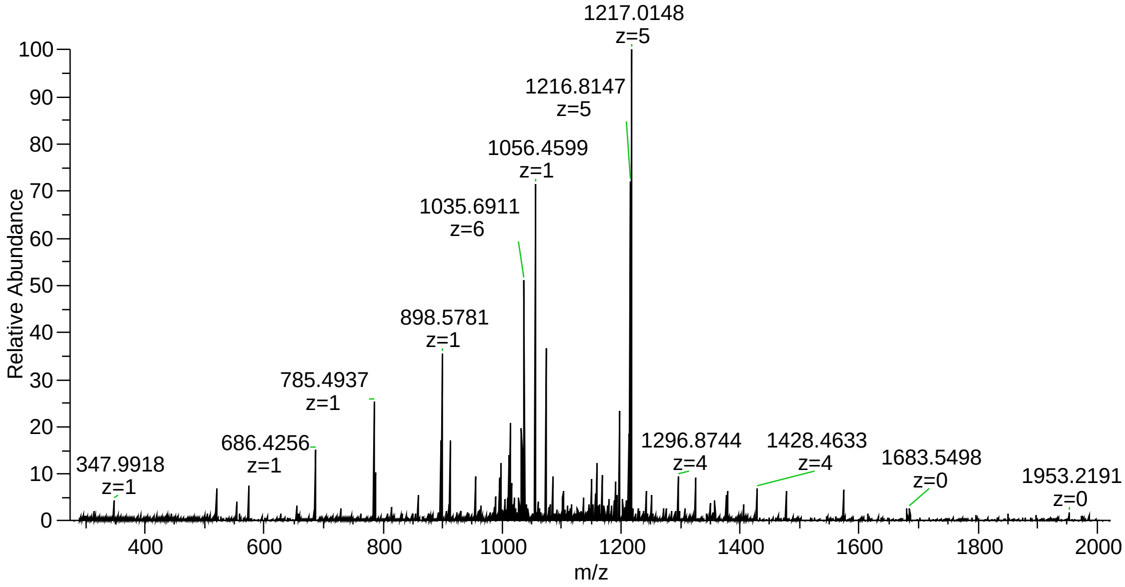

| Fragment | Observed mass (monoisotopic) [Da] | Theoretical mass (monoisotopic) [Da] | Mass difference [Da] | Mass difference [ppm] |
|---|---|---|---|---|
| B6 | 685.4206 | 685.4197 | 0.0009 | 1.3656 |
| B7 | 784.4873 | 784.4881 | -0.0007 | -0.9216 |
| B8 | 897.5724 | 897.5721 | 0.0003 | 0.3454 |
| B9 | 954.5918 | 954.5936 | -0.0017 | -1.8291 |
| B38 | 4120.0951 | 4120.1080 | -0.0129 | -3.1310 |
| B55 | 5978.9244 | 5978.9737 | -0.0493 | -8.2533 |
| B55 | 5978.9260 | 5978.9737 | -0.0477 | -7.9800 |
| B56 | 6078.0274 | 6078.0421 | -0.0147 | -2.4166 |
| B56 | 6078.0407 | 6078.0421 | -0.0014 | -0.2325 |

Protein name: Entericidin B (Uniprot accession no. B0V4V9)
Charge state: +4
Observed monoisotopic mass: 3903.1785 Da
Sequence: MMKKVLVASVMVAFVLTGCNTFKGFGQDVSKAGDAVTNTAQKTENKM
Signal peptide cleaved 1-18
Lipid attached to the N-terminal cysteine: lipid-CNTFKGFGQDVSKAGDAVTNTAQKTENKM

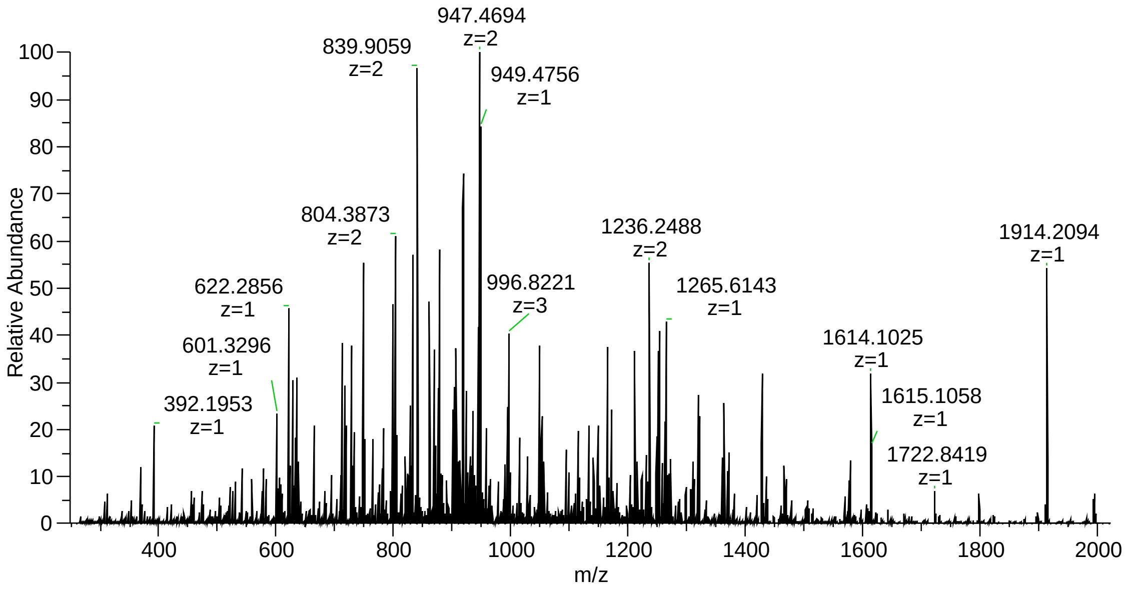


| Observed mass (monoisotopic) [Da] | Theoretical mass (monoisotopic) [Da] | Charge | Fragment | Mass difference [Da] | Mass difference [ppm] |
|---|---|---|---|---|---|
| 392.1953 | 392.1962 | 1 | y3 | -0.0009 | -2.2948 |
| 525.7653 | 525.7660 | 2 | y9 | -0.0014 | -0.8559 |
| 582.7869 | 582.7875 | 2 | y10 | -0.0012 | -0.7722 |

| | | | | | |
|---|---|---|---|---|---|
| 602.9712 | 602.9720 | 3 | y17 | -0.0024 | -0.4975 |
| 622.2856 | 622.2865 | 1 | y5 | -0.0009 | -1.4463 |
| 633.3107 | 633.3114 | 2 | y11 | -0.0014 | -0.7106 |
| 665.0046 | 665.0055 | 3 | y19 | -0.0027 | -0.4511 |
| 682.8443 | 682.8456 | 2 | y12 | -0.0026 | -0.6590 |
| 703.3470 | 703.3478 | 3 | y20 | -0.0024 | -0.4265 |
| 718.3632 | 718.3641 | 2 | y13 | -0.0018 | -0.6264 |
| 747.6178 | 747.6186 | 4 | y28 | -0.0032 | -0.3010 |
| 750.3803 | 750.3815 | 1 | y6 | -0.0012 | -1.1994 |
| 765.0400 | 765.0411 | 3 | y22 | -0.0033 | -0.3921 |
| 775.8759 | 775.8776 | 2 | y14 | -0.0034 | -0.5800 |
| 804.3873 | 804.3883 | 2 | y15 | -0.0020 | -0.5594 |
| 839.9059 | 839.9069 | 2 | y16 | -0.0020 | -0.5358 |
| 875.7684 | 875.7694 | 3 | y25 | -0.0030 | -0.3426 |
| 878.4387 | 878.4400 | 1 | y7 | -0.0013 | -1.0245 |
| 903.9538 | 903.9544 | 2 | y17 | -0.0012 | -0.4978 |

***E. cloacae* S11**

Protein name: DNA-binding protein (Uniprot accession no. A0A094ZZI7)
Charge state: +10
Observed monoisotopic mass: 9499.1592 Da
Sequence:
MNKTQLIDVIADKADLSKVQAKAALESTLAAITESLKEGDAVQLVGFGTFKVNHRAERTGRNPQTGKEIKIAAANVPAFVSGKALKDAVK

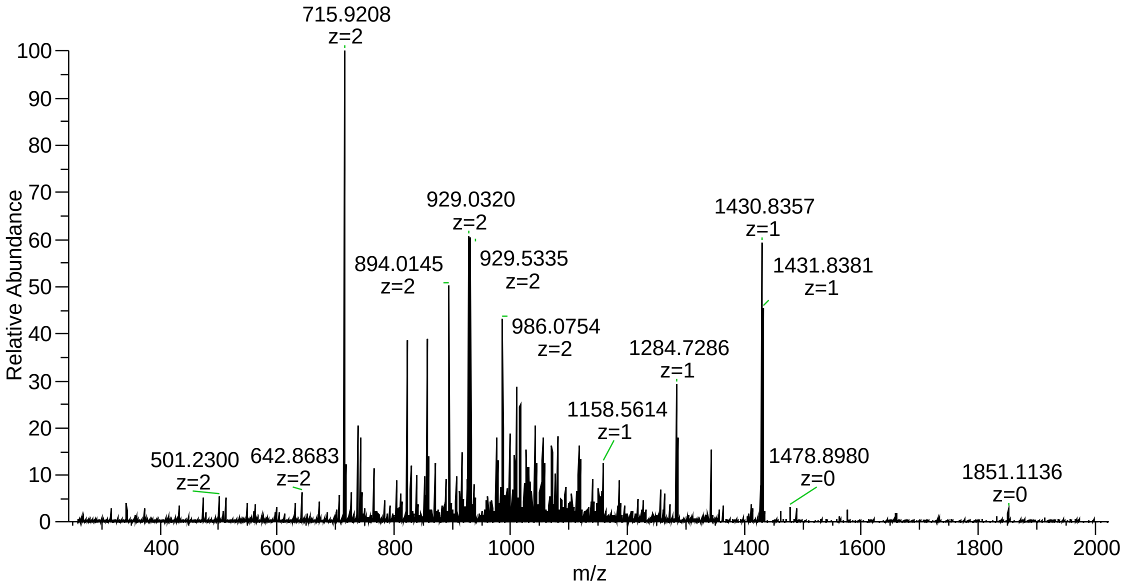


| Observed mass (monoisotopic) [Da] | Theoretical mass (monoisotopic) [Da] | Charge | Fragment | Mass difference [Da] | Mass difference [ppm] |
|---|---|---|---|---|---|
| 432.2445 | 432.2453 | 4 | y4 | -0.0032 | -1.8508 |
| 475.2327 | 475.2333 | 1 | b4 | -0.0006 | -1.2625 |
| 477.6166 | 477.6170 | 3 | y14 | -0.0012 | -0.8375 |
| 508.8079 | 508.8086 | 2 | y10 | -0.0014 | -1.3758 |
| 560.3397 | 560.3402 | 1 | y5 | -0.0005 | -0.8923 |
| 603.2910 | 603.2919 | 1 | b5 | -0.0009 | -1.4918 |
| 631.8757 | 631.8770 | 2 | y12 | -0.0026 | -2.0574 |
| 673.4230 | 673.4243 | 1 | y6 | -0.0013 | -1.9304 |
| 715.9208 | 715.9219 | 2 | y14 | -0.0022 | -1.5365 |
| 737.7781 | 737.7790 | 3 | y22 | -0.0027 | -1.2199 |
| 744.4601 | 744.4614 | 1 | y7 | -0.0013 | -1.7462 |
| 765.4546 | 765.4561 | 2 | y15 | -0.0030 | -1.9596 |
| 771.4194 | 771.4214 | 2 | b14 | -0.0040 | -2.5926 |

| | | | | | |
|---|---|---|---|---|---|
| 792.6863 | 792.6891 | 4 | b30 | -0.0112 | -3.5323 |
| 804.4473 | 804.4491 | 3 | b22 | -0.0054 | -2.2376 |
| 810.4465 | 810.4483 | 4 | b31 | -0.0072 | -2.2210 |
| 822.4765 | 822.4776 | 2 | y16 | -0.0022 | -1.3374 |
| 851.8063 | 851.8071 | 3 | b24 | -0.0024 | -0.9392 |
| 854.1449 | 854.1459 | 6 | y48 | -0.0060 | -1.1708 |
| 857.9950 | 857.9962 | 2 | y17 | -0.0024 | -1.3986 |
| 889.5009 | 889.5018 | 3 | b25 | -0.0027 | -1.0118 |
| 893.5133 | 893.5147 | 2 | y18 | -0.0028 | -1.5668 |
| 907.3909 | 907.3942 | 9 | y78 | -0.0297 | -3.6368 |
| 911.1706 | 911.1716 | 6 | y52 | -0.0060 | -1.0975 |
| 915.9125 | 915.9135 | 5 | y43 | -0.0050 | -1.0918 |
| 929.0320 | 929.0333 | 2 | y19 | -0.0026 | -1.3993 |
| 932.6777 | 932.6787 | 6 | y53 | -0.0060 | -1.0722 |
| 956.7308 | 956.7315 | 5 | y45 | -0.0035 | -0.7317 |
| 976.5450 | 976.5451 | 5 | y46 | -0.0005 | -0.1024 |
| 983.0321 | 983.0370 | 8 | b74 | -0.0392 | -4.9846 |
| 985.5736 | 985.5753 | 2 | y20 | -0.0034 | -1.7249 |
| 999.1596 | 999.1619 | 5 | y47 | -0.0115 | -2.3019 |
| 1009.6728 | 1009.6759 | 8 | b76 | -0.0248 | -3.0703 |
| 1015.0033 | 1015.0072 | 9 | y87 | -0.0351 | -3.8423 |
| 1024.7717 | 1024.7737 | 5 | y48 | -0.0100 | -1.9517 |
| 1025.7272 | 1025.7289 | 6 | y58 | -0.0102 | -1.6574 |
| 1029.2375 | 1029.2400 | 9 | y88 | -0.0225 | -2.4290 |
| 1032.9018 | 1032.9040 | 3 | b29 | -0.0066 | -2.1299 |
| 1042.1463 | 1042.1480 | 7 | y70 | -0.0119 | -1.6312 |
| 1053.5652 | 1053.5674 | 4 | b40 | -0.0088 | -2.0881 |
| 1056.4165 | 1056.4158 | 6 | y60 | 0.0042 | 0.6626 |
| 1068.2537 | 1068.2553 | 6 | y61 | -0.0096 | -1.4978 |
| 1071.3253 | 1071.3267 | 4 | b41 | -0.0056 | -1.3068 |
| 1076.7270 | 1076.7276 | 7 | b70 | -0.0042 | -0.5572 |
| 1080.2600 | 1080.2620 | 3 | b31 | -0.0060 | -1.8514 |
| 1118.4460 | 1118.4493 | 6 | y64 | -0.0198 | -2.9505 |
| 1223.6620 | 1223.6642 | 3 | b35 | -0.0066 | -1.7979 |
| 1227.6728 | 1227.6766 | 1 | b11 | -0.0038 | -3.0953 |
| 1261.3575 | 1261.3588 | 3 | b36 | -0.0039 | -1.0306 |
| 1342.7035 | 1342.7035 | 1 | b12 | 0.0000 | 0.0000 |
| 1428.0990 | 1428.0999 | 3 | b41 | -0.0027 | -0.6302 |
| 1430.8357 | 1430.8366 | 1 | y14 | -0.0009 | -0.6290 |
| 1461.1238 | 1461.1227 | 3 | b42 | 0.0033 | 0.7528 |

Protein name: CsbD family protein (Uniprot accession no. A0A0M2G4R3)
Charge state: +7
Observed monoisotopic mass: 8322.0209 Da
Sequence:
MNKDEIGGNWKQFKGKAKEQWGKLTDDDMTVIEGKRDQLVGKIQERYGYEKDQAENEVKDWETRN
DYRW

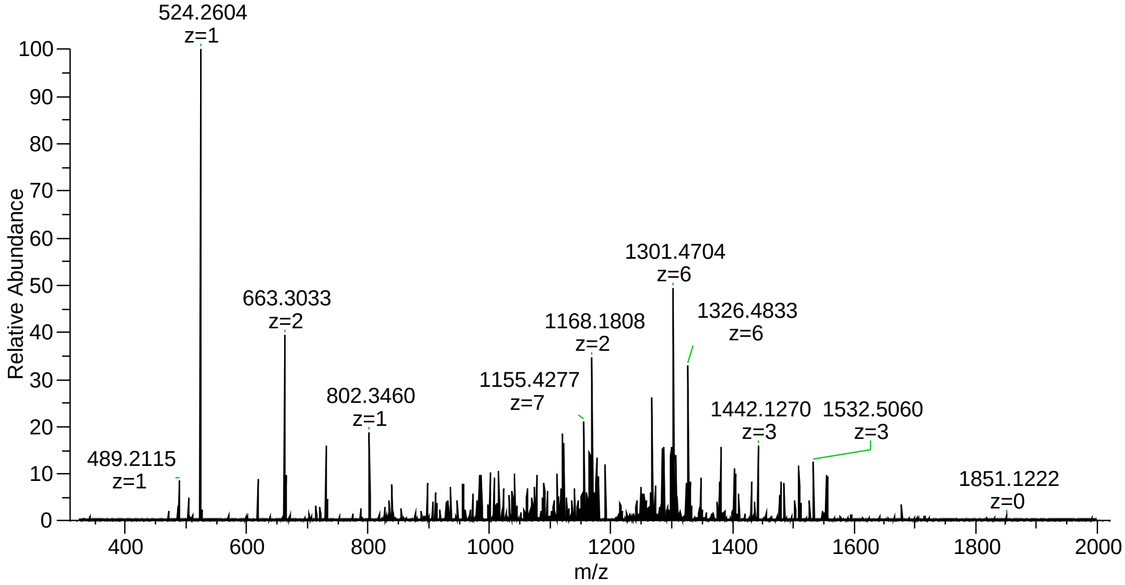


| Observed mass (monoisotopic) [Da] | Theoretical mass (monoisotopic) [Da] | Charge | Fragment | Mass difference [Da] | Mass difference [ppm] |
|---|---|---|---|---|---|
| 489.2115 | 489.2126 | 1 | b4 | -0.0011 | -2.2485 |
| 505.7428 | 505.7438 | 2 | y6 | -0.0020 | -1.9773 |
| 524.2604 | 524.2616 | 1 | y3 | -0.0012 | -2.2889 |
| 618.2536 | 618.2552 | 1 | b5 | -0.0016 | -2.5879 |
| 663.3033 | 663.3047 | 2 | y9 | -0.0028 | -2.1106 |
| 720.8162 | 720.8182 | 2 | y10 | -0.0040 | -2.7746 |
| 731.3375 | 731.3393 | 1 | b6 | -0.0018 | -2.4612 |
| 753.3292 | 753.3315 | 1 | b5 | -0.0023 | -3.0531 |
| 774.8642 | 774.8668 | 2 | b13 | -0.0052 | -3.3554 |
| 788.3587 | 788.3607 | 1 | b7 | -0.0020 | -2.5369 |
| 834.3975 | 834.3999 | 2 | y12 | -0.0048 | -2.8763 |
| 897.1245 | 897.1271 | 3 | b23 | -0.0078 | -2.8981 |
| 931.4707 | 931.4725 | 2 | b16 | -0.0036 | -1.9324 |
| 934.8195 | 934.8218 | 3 | b24 | -0.0069 | -2.4604 |
| 955.9406 | 955.9426 | 2 | y14 | -0.0040 | -2.0922 |

| | | | | | |
|---|---|---|---|---|---|
| 1006.8435 | 1006.8467 | 3 | b26 | -0.0096 | -3.1782 |
| 1020.4618 | 1020.4639 | 2 | y15 | -0.0042 | -2.0579 |
| 1031.0361 | 1031.0386 | 2 | b18 | -0.0050 | -2.4247 |
| 1050.8115 | 1050.8149 | 3 | y24 | -0.0102 | -3.2356 |
| 1055.9795 | 1055.9825 | 2 | y16 | -0.0060 | -2.8410 |
| 1083.5291 | 1083.5313 | 3 | b28 | -0.0066 | -2.0304 |
| 1095.5593 | 1095.5599 | 2 | b19 | -0.0012 | -0.5477 |
| 1120.0118 | 1120.0098 | 2 | y17 | 0.0040 | 1.7857 |
| 1126.3001 | 1126.3023 | 4 | y36 | -0.0088 | -1.9533 |
| 1154.8540 | 1154.8575 | 7 | y67 | -0.0245 | -3.0307 |
| 1217.8101 | 1217.8125 | 5 | b52 | -0.0120 | -1.9707 |
| 1236.8598 | 1236.8629 | 4 | y40 | -0.0124 | -2.5063 |
| 1241.5690 | 1241.5727 | 2 | y19 | -0.0074 | -2.9801 |
| 1252.6267 | 1252.6288 | 2 | b21 | -0.0042 | -1.6765 |
| 1256.7851 | 1256.7876 | 6 | y62 | -0.0150 | -1.9892 |
| 1266.2865 | 1266.2912 | 6 | y63 | -0.0282 | -3.7116 |
| 1269.6217 | 1269.6231 | 4 | y41 | -0.0056 | -1.1027 |
| 1273.5964 | 1273.5994 | 1 | b11 | -0.0030 | -2.3555 |
| 1285.1352 | 1285.1385 | 6 | y64 | -0.0198 | -2.5678 |
| 1300.8019 | 1300.8041 | 6 | b66 | -0.0132 | -1.6913 |
| 1306.6447 | 1306.6456 | 6 | y65 | -0.0054 | -0.6888 |
| 1325.8160 | 1325.8168 | 6 | y66 | -0.0048 | -0.6034 |
| 1330.2500 | 1330.2515 | 5 | y55 | -0.0075 | -1.1276 |
| 1347.1623 | 1347.1659 | 6 | y67 | -0.0216 | -2.6723 |
| 1349.3193 | 1349.3191 | 3 | y32 | 0.0006 | 0.1482 |
| 1409.4257 | 1409.4262 | 4 | y46 | -0.0020 | -0.3548 |
| 1676.8185 | 1676.8213 | 1 | b14 | -0.0028 | -1.6698 |

Protein name: UPF0391 membrane protein SAMEA2054040_04753 (Uniprot accession no. A0A094XPL9)
Charge state: +5
Observed monoisotopic mass: 5687.2876 Da
Sequence: fMFRWGIIFLVIALIAAALGFGGLAGTAAWAAKIVFVVGIILFLVSLFTGRRRP

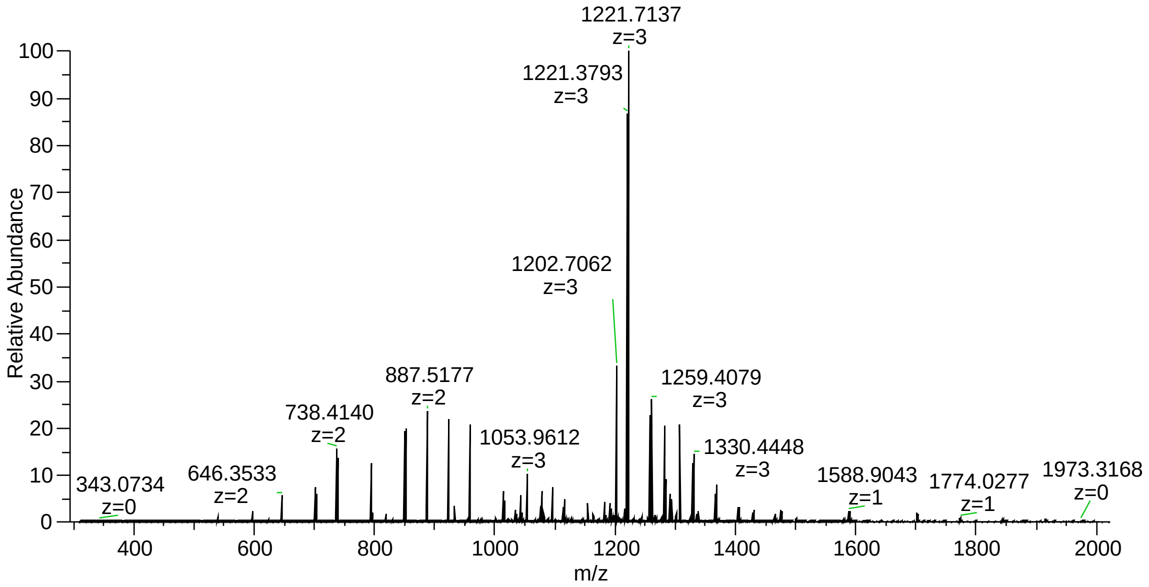


| Fragment | Observed mass (monoisotopic) [Da] | Theoretical mass (monoisotopic) [Da] | Mass difference [Da] | Mass difference [ppm] |
|---|---|---|---|---|
| B6 | 818.3909 | 818.3898 | 0.0011 | 1.3771 |
| B7 | 931.4730 | 931.4738 | -0.0008 | -0.8578 |
| B8 | 1078.5418 | 1078.5422 | -0.0004 | -0.4015 |
| B9 | 1191.6255 | 1191.6263 | -0.0008 | -0.6571 |
| B10 | 1290.6937 | 1290.6947 | -0.0010 | -0.7663 |
| B10 | 1290.6972 | 1290.6947 | 0.0025 | 1.9571 |
| B11 | 1403.7746 | 1403.7788 | -0.0042 | -2.9948 |
| B11 | 1403.7814 | 1403.7788 | 0.0026 | 1.8628 |
| B12 | 1474.8163 | 1474.8159 | 0.0004 | 0.2990 |
| B12 | 1474.8174 | 1474.8159 | 0.0016 | 1.0659 |
| B13 | 1587.8932 | 1587.8999 | -0.0067 | -4.2263 |
| B13 | 1587.9029 | 1587.8999 | 0.0030 | 1.8628 |
| B14 | 1700.9814 | 1700.9840 | -0.0026 | -1.5285 |
| B15 | 1772.0213 | 1772.0211 | 0.0002 | 0.1185 |
| B15 | 1772.0239 | 1772.0211 | 0.0028 | 1.6044 |

| | | | | |
|---|---|---|---|---|
| B16 | 1843.0459 | 1843.0582 | -0.0124 | -6.7024 |
| B17 | 1914.0915 | 1914.0953 | -0.0038 | -1.9910 |
| B18 | 2027.1712 | 2027.1794 | -0.0082 | -4.0366 |
| B19 | 2084.2011 | 2084.2008 | 0.0003 | 0.1420 |
| B20 | 2231.2551 | 2231.2692 | -0.0142 | -6.3439 |
| B22 | 2345.3062 | 2345.3122 | -0.0060 | -2.5485 |
| B23 | 2458.3709 | 2458.3962 | -0.0253 | -10.3092 |
| B24 | 2529.4142 | 2529.4333 | -0.0191 | -7.5539 |
| B35 | 3645.0714 | 3645.0461 | 0.0254 | 6.9631 |
| Y15 | 1787.0897 | 1787.0934 | -0.0037 | -2.0693 |
| Y17 | 1943.1847 | 1943.1832 | 0.0014 | 0.7395 |
| Y18 | 2042.2440 | 2042.2516 | -0.0076 | -3.7189 |
| Y22 | 2529.5684 | 2529.5675 | 0.0009 | 0.3744 |
| Y23 | 2600.5821 | 2600.6046 | -0.0225 | -8.6345 |
| Y24 | 2671.6311 | 2671.6417 | -0.0106 | -3.9710 |
| Y25 | 2857.7243 | 2857.7210 | 0.0033 | 1.1457 |
| Y26 | 2928.7468 | 2928.7581 | -0.0113 | -3.8692 |
| Y26 | 2928.7570 | 2928.7581 | -0.0011 | -0.3869 |
| Y27 | 2999.7880 | 2999.7952 | -0.0073 | -2.4232 |
| Y27 | 2999.7917 | 2999.7952 | -0.0035 | -1.1697 |
| Y28 | 3100.8153 | 3100.8429 | -0.0276 | -8.9031 |
| Y29 | 3157.8527 | 3157.8644 | -0.0117 | -3.7031 |
| Y29 | 3157.8599 | 3157.8644 | -0.0044 | -1.4032 |
| Y30 | 3228.8829 | 3228.9015 | -0.0186 | -5.7589 |
| Y34 | 3603.0661 | 3603.0969 | -0.0308 | -8.5352 |
| Y35 | 3660.1084 | 3660.1183 | -0.0099 | -2.7125 |
| Y37 | 3844.2189 | 3844.2395 | -0.0206 | -5.3701 |
| Y38 | 3915.2697 | 3915.2766 | -0.0069 | -1.7562 |
| Y39 | 3986.2815 | 3986.3137 | -0.0323 | -8.0932 |
| Y40 | 4099.3685 | 4099.3978 | -0.0293 | -7.1430 |
| Y41 | 4212.4563 | 4212.4818 | -0.0256 | -6.0720 |

Protein name: 50S ribosomal protein L29 (Uniprot accession no. A0A2T4XV45)
Charge state: +7
Observed monoisotopic mass: 7238.0086 Da
Sequence:
MKAKELREKSVEELNAELLNLLREQFNLRMQAASGQLQQTHLLKQVRRNVARVKTLLTQKAGA

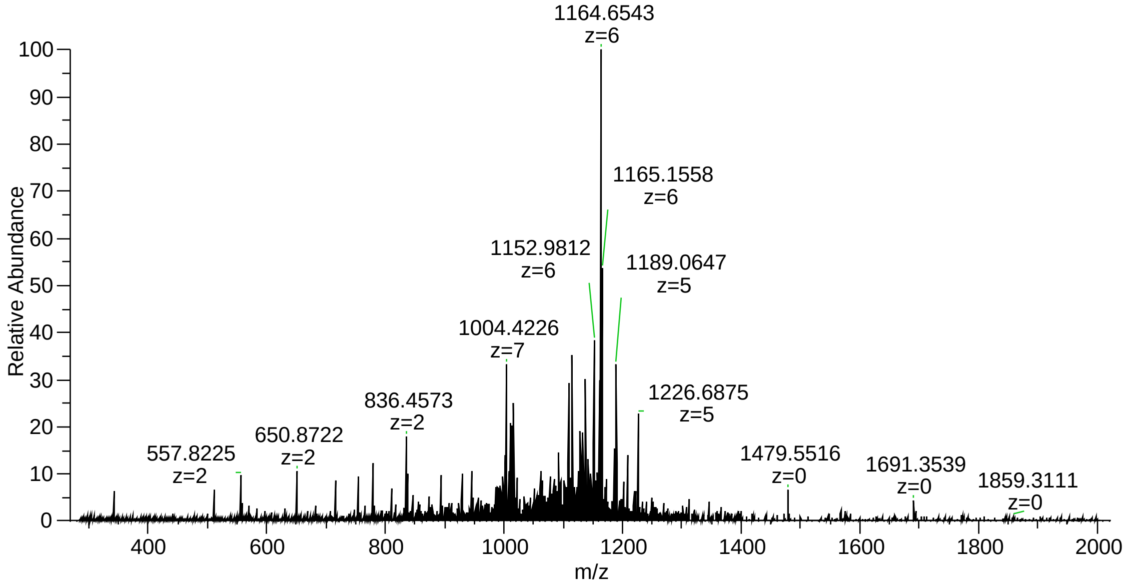


| Fragment | Observed mass (monoisotopic) [Da] | Theoretical mass (monoisotopic) [Da] | Mass difference [Da] | Mass difference [ppm] |
|---|---|---|---|---|
| B9 | 1113.6275 | 1113.6328 | -0.0054 | -4.8113 |
| B9 | 1113.6299 | 1113.6328 | -0.0029 | -2.5987 |
| B12 | 1428.7783 | 1428.7759 | 0.0025 | 1.7162 |
| B13 | 1557.8142 | 1557.8185 | -0.0043 | -2.7404 |
| B14 | 1670.9064 | 1670.9025 | 0.0039 | 2.3209 |
| B16 | 1855.9661 | 1855.9826 | -0.0164 | -8.8379 |
| B17 | 1985.0148 | 1985.0251 | -0.0104 | -5.2211 |
| B62 | 7148.9156 | 7148.9771 | -0.0615 | -8.6037 |
| Y47 | 5382.0189 | 5382.0422 | -0.0233 | -4.3318 |
| Y48 | 5453.0588 | 5453.0793 | -0.0205 | -3.7623 |
| Y49 | 5567.1257 | 5567.1222 | 0.0035 | 0.6291 |
| Y51 | 5809.2177 | 5809.2489 | -0.0312 | -5.3701 |
| Y52 | 5938.2590 | 5938.2915 | -0.0324 | -5.4640 |
| Y53 | 6037.3280 | 6037.3599 | -0.0319 | -5.2882 |
| Y54 | 6124.3528 | 6124.3919 | -0.0391 | -6.3908 |

| | | | | |
|---|---|---|---|---|
| Y58 | 6650.6697 | 6650.7146 | -0.0450 | -6.7633 |
| Y59 | 6779.7214 | 6779.7572 | -0.0359 | -5.2905 |
| Y60 | 6907.8102 | 6907.8522 | -0.0420 | -6.0844 |
| Y61 | 6978.8647 | 6978.8893 | -0.0246 | -3.5304 |

Protein name: DUF1471 domain-containing protein (Uniprot accession no. A0A176X7A3)
Charge state: +8
Observed monoisotopic mass: 7667.8618 Da
Sequence:
ADMISKDEAHHFKLEYLGNVSVGASGGQISSPSDLHNKLSKLADEKGGKYYVIIAAREHGPNFQAVAEVF
K

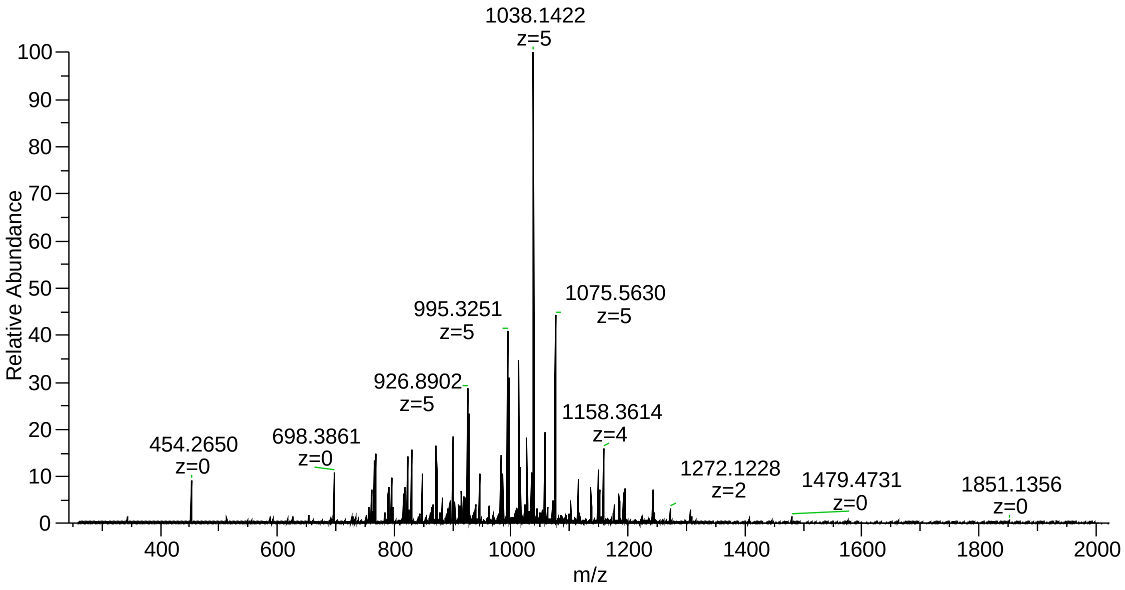


| Fragment | Observed mass (monoisotopic) [Da] | Theoretical mass (monoisotopic) [Da] | Mass difference [Da] | Mass difference [ppm] |
|---|---|---|---|---|
| B11 | 1234.5386 | 1234.5401 | -0.0014 | -1.1672 |
| B17 | 2027.9663 | 2027.9775 | -0.0112 | -5.5104 |
| B18 | 2084.9866 | 2084.9989 | -0.0123 | -5.8911 |
| B19 | 2199.0238 | 2199.0419 | -0.0181 | -8.2259 |
| B19 | 2199.0402 | 2199.0419 | -0.0016 | -0.7399 |
| B20 | 2298.1068 | 2298.1103 | -0.0035 | -1.5139 |
| B20 | 2298.1121 | 2298.1103 | 0.0019 | 0.8189 |
| B21 | 2385.1302 | 2385.1423 | -0.0121 | -5.0852 |

| | | | | |
|---|---|---|---|---|
| B21 | 2385.1381 | 2385.1423 | -0.0042 | -1.7513 |
| B22 | 2484.1862 | 2484.2107 | -0.0245 | -9.8442 |
| B22 | 2484.1983 | 2484.2107 | -0.0124 | -4.9879 |
| B23 | 2541.2108 | 2541.2322 | -0.0214 | -8.4069 |
| B24 | 2612.2625 | 2612.2693 | -0.0067 | -2.5748 |
| B24 | 2612.2630 | 2612.2693 | -0.0063 | -2.4128 |
| B25 | 2699.2873 | 2699.3013 | -0.0140 | -5.1999 |
| B28 | 2941.3874 | 2941.4028 | -0.0154 | -5.2220 |
| Y7 | 762.4261 | 762.4276 | -0.0014 | -1.8953 |
| Y9 | 1022.5212 | 1037.5546 | -0.0057 | -5.4499 |
| Y12 | 1305.6717 | 1305.6717 | 0.0000 | -0.0260 |
| Y16 | 1798.9056 | 1798.9114 | -0.0058 | -3.2181 |
| Y17 | 1869.9359 | 1869.9485 | -0.0126 | -6.7611 |
| Y18 | 1983.0188 | 1983.0326 | -0.0138 | -6.9641 |
| Y19 | 2096.1054 | 2096.1167 | -0.0112 | -5.3623 |
| Y43 | 4726.4629 | 4741.4867 | 0.0039 | 0.8323 |
| Y43 | 4726.4916 | 4741.4867 | 0.0326 | 6.8743 |
| Y45 | 4911.5468 | 4926.5667 | 0.0077 | 1.5728 |
| Y46 | 4968.5748 | 4983.5882 | 0.0143 | 2.8793 |
| Y47 | 5055.5979 | 5070.6202 | 0.0054 | 1.0682 |
| Y47 | 5055.5988 | 5070.6202 | 0.0063 | 1.2356 |
| Y48 | 5126.6100 | 5141.6573 | -0.0196 | -3.8111 |
| Y48 | 5126.6446 | 5141.6573 | 0.0150 | 2.9168 |
| Y49 | 5183.6706 | 5198.6788 | 0.0195 | 3.7516 |
| Y49 | 5183.6744 | 5198.6788 | 0.0233 | 4.4831 |
| Y50 | 5282.7108 | 5297.7472 | -0.0087 | -1.6434 |
| Y50 | 5282.7279 | 5297.7472 | 0.0084 | 1.5851 |
| Y51 | 5369.7320 | 5384.7792 | -0.0195 | -3.6218 |
| Y51 | 5369.7570 | 5384.7792 | 0.0055 | 1.0205 |
| Y52 | 5468.8225 | 5483.8476 | 0.0026 | 0.4742 |
